# Xiong Fu Powder Regulates the Intestinal Microenvironment to Protect Bones Against Destruction in Collagen-Induced Arthritis Rat Models

**DOI:** 10.3389/fcimb.2022.854940

**Published:** 2022-07-01

**Authors:** Xiaoyu Xi, Qinbin Ye, Xiaoya Li, Xiangchen Lu, Danping Fan, Ya Xia, Cheng Xiao

**Affiliations:** ^1^ School of Traditional Chinese Medicine, Beijing University of Chinese Medicine, Beijing, China; ^2^ The Institute of Medicinal Plant Development, Chinese Academy of Medical Sciences/Peking Union Medical College, Beijing, China; ^3^ Pinggu Hospital, Beijing Traditional Chinese Medicine Hospital, Beijing, China; ^4^ Institute of Clinical Medical Sciences, China–Japan Friendship Hospital, Beijing, China; ^5^ Graduate School of Peking Union Medical College, Chinese Academy of Medical Sciences/Peking Union Medical College, Beijing, China; ^6^ Department of Emergency, China–Japan Friendship Hospital, Beijing, China

**Keywords:** Xiong Fu powder, collagen-induced arthritis, bone destruction, intestinal microenvironment, *Lactobacillus*

## Abstract

**Background:**

Changes in the intestinal microenvironment affected bone destruction in rheumatoid arthritis (RA), and spleen deficiency (SD) was closely related to the intestinal microenvironment. In this study, we aimed to explore the aggravation of SD on collagen-induced arthritis (CIA) and the bone protection of compound Xiong Fu powder (XFP) on CIA with SD (SD-CIA) based on the intestinal microenvironment.

**Method:**

An SD-CIA rat model was established using *Rheum officinale* Baill. decoction combined with CIA and then treated with XFP. The aggravating action of SD on CIA rats and the efficacy of XFP were evaluated using AI scores, H&E staining of the joint, and level of serum anti–collagen type II antibody (Col II Ab). Bone destruction was assessed by micro-CT and TRACP staining. In addition, flow cytometry, qRT-PCR, and ELISA were used to evaluate gut mucosal immunity. Moreover, metagenomic sequencing was used to determine the distribution and function of the gut microbiota.

**Results:**

Compared with that in CIA rats, bone destruction in SD-CIA rats was aggravated, as manifested by increased AI scores, more severe joint pathological changes and radiological damage, and increased number of osteoclasts (OCs) in the ankle joint. Meanwhile, the proportion of Tregs/Th17 cells was biased toward Th17 cells in Peyer’s patches. Furthermore, the gene levels of *TNF-α, IL-1β, IL-6, and IL-17* were increased. In contrast, the expression of *IL-10* and sIgA was decreased in the jejunum and ileum. XFP treatment improved bone damage and intestinal mucosal immune disorders compared with the SD-CIA group. In addition, the distribution and function of the gut microbiota were altered in the SD-CIA group. After XFP treatment, the community and function of the gut microbiota were regulated, manifested as increased abundance of several *Lactobacillus* species, such as *L. acidophilus*, which regulates the intestinal Tregs/Th17 cells and quorum sensing pathways, followed by promoting probiotic adhesion to the intestines.

**Conclusion:**

SD can aggravate bone destruction in CIA rats. Compound XFP may attenuate bone destruction in SD-CIA rats by regulating the intestinal microenvironment. One of the mechanisms is the cross-talk between sIgA secretion regulated by intestinal mucosal Tregs and Th17 cells and adhesion of *Lactobacillus* mediated by quorum sensing.

## Introduction

Rheumatoid arthritis (RA) is a common chronic inflammatory systemic autoimmune disease characterized by synovial inflammation, joint swelling, and the destruction of bone or cartilage (McInnes and Schett, 2011). Focal bone destruction in inflammatory joints is a hallmark of RA and can lead to functional disability and the development of secondary osteoporosis (McInnes and Schett, 2011). Previous studies have demonstrated that the balance between T regulatory cells (Tregs) and T helper 17 (Th17) lymphocytes affects osteoclast (OC)–mediated bone destruction (Koyama and Tanaka, 2016; Xu et al., 2016; Zhao et al., 2018). In addition, disorders of the intestinal microenvironment, primarily composed of intestinal mucosal immunity and the gut microbiota, can affect the systemic immune system and lead to further bone destruction in RA (Xu C et al., 2020; Xu Z et al., 2020).

In the intestinal microenvironment, intestinal mucosal immunity and the gut microbiota exhibit a bidirectional regulatory effect. It is well understood that strains from *Lactobacillus*, such as *L. acidophilus* (Yanagihara et al., 2014; Chen et al., 2015; Li et al., 2019; Pan et al., 2019), *L. murinus* (Wilck et al., 2017; Bernard-Raichon et al., 2021), *L. johnsonii* (Lau et al., 2011; Zheng et al., 2021), and *L. reuteri* (Mu et al., 2017), display strong immune regulation ability, especially with respect to Tregs and Th17 cells. On the other hand, enteric-related tissues such as mesenteric lymph nodes, Peyer’s patches (PPs) and lamina propria are rich in immune cells to resist attack from intestinal pathogens. Among them, secretory immunoglobulin A (sIgA), indirectly regulated by Tregs and Th17 cells, plays an important role in maintaining the intestinal barrier. Pan et al. found that by administering *L. casei* to adjuvant-induced arthritis (AIA) rats, the abundance of *L. acidophilus* was significantly increased, accompanied by downregulation of interleukin-17 (IL-17) and reduced destruction of articular bone (Pan et al., 2019). Our previous study also demonstrated that human umbilical mesenchymal stem cells (HUMSCs) upregulate ileal sIgA secretion and improve intestinal microflora abnormalities in collagen-induced arthritis (CIA) rats, which slowed the process of bone destruction (Li et al., 2020). Therefore, by maintaining intestinal microenvironment homeostasis, the systemic immune status can be improved, and bone protection can be exerted in RA.

According to the theory of traditional Chinese medicine (TCM), RA is a Chinese arthralgia syndrome, and spleen deficiency (SD) is one of the basic pathogeneses of RA. Patients with SD exhibit decreased metabolic levels and immune responses due to poor absorption function. Interestingly, many probiotics, such as *Lactobacilli*, are involved in lipid, carbohydrate, and amino acid metabolism (Drissi et al., 2017), and changes in their abundance and function affect the absorption of nutrients, which coincides with the cause of SD syndrome. Our previous studies have shown that a more obvious inflammatory response and degeneration of cartilage were observed in CIA rats with SD (SD-CIA) compared with CIA rats. In addition, mucosal immune function was downregulated (Xiao et al., 2003). The ancient prescription of Xiong Fu powder (XFP) is from the book named *Pu Ji Ben Shi Fang* of the Southern Song Dynasty, and its main function is to treat “a patient who had a weak spleen and stomach, invaded by wind-cold-dampness, and the defensive Qi does not cover the muscles”, which is, in fact, RA with SD. Our previous study found that XFP had a marked effect on SD-CIA (Xiao et al., 2007). XFP consists of 10 Chinese herbs, including *Astragalus mongholicus* Bunge., *Atractylodes macrocephala* Koidz., *Saposhnikovia divaricata* (Turcz. Ex Ledeb.) Schischk., and *Glycyrrhiza glabra* L. Interestingly, various herbs of XFP exhibited the ability to regulate the intestinal flora (Shi et al., 2019; Zou et al., 2021). However, it has not been reported whether SD aggravates CIA through the intestinal microenvironment or whether XFP can play an osteoprotective role by regulating intestinal microenvironment homeostasis.

In this study, the SD-CIA model was established by *Rheum officinale* Baill. purgation combined with CIA and treated with XFP, the mechanism by which XFP improved SD-CIA was researched with respect to the intestinal microenvironment. This study has an important implication for the selection of therapeutic targets and evaluation of the therapeutic effect of Chinese arthralgia syndrome treated from the spleen.

## Materials and Methods

### Animals

A total of 50 male Sprague–Dawley rats (210 ± 10 g) were purchased from the National Institutes for Food and Drug Control [animal licence number: SCXK (Beijing) 2019-0017]. Rats were housed in the Experimental Animal Centre of the Institute of Clinical Medical Sciences, China–Japan Friendship Hospital [Experiment Animal Centre licence number: SYXK (Beijing) 2016-0043]. In a standard light/dark cycle (6:00–18:00 light phase), the rats were maintained at a constant temperature of 23°C ( ± 2°C) with free access to standard rodent feed and water in a specific pathogen-free animal laboratory. All experimental procedures were reviewed, approved, and directed by the Institute of Clinical Medical Science, China–Japan Friendship Hospital (Ethics No. 190104).

### Preparation of Decoction From *Rheum officinale* Baill. and XFP

Decoction from *Rheum officinale* Baill. (Polygonaceae, Rhei radix et rhizoma) (Beijing Tongrentang Co., Ltd., Beijing, China) was used to induce the SD rat model. In detail, 1 kg of *Rheum officinale* Baill. was made into powder and dissolved in 5 L of pure water followed by ultrasonication for 1 h. Then, the solution was filtered with four layers of gauze and centrifuged at 2,500 rpm for 5 min. The supernatant was collected and concentrated to 500 ml by rotary evaporation and stored at 4°C. XFP is composed of the following herbs: *Astragalus mongholicus* Bunge. (15 g, Fabaceae, Astragali radix), *Atractylodes macrocephala* Koidz. (15 g, Asteraceae, Atractylodis macrocephalae rhizome), *Saposhnikovia divaricata* (Turcz. Ex Ledeb.) Schischk. (10 g, Apiaceae, Saposhnikoviae radix), *Glycyrrhiza glabra* L. (10 g, Fabaceae, Glycyrrhizae radix et rhizome), *Rehmannia glutinosa* (Gaertn.) DC. (15 g, Scrophulariaceae, Rehmanniae radix praeparata), *Angelica sinensis* (Oliv.) Diels. (10 g, Apiaceae, Angelicae sinensis radix), *Ligusticum chuanxiong* Hort. (10 g, Apiaceae, Chuanxiong rhizoma), *Bupleurum chinense* DC. (10 g, Apiaceae, Bupleuri radix), *Aconitum carmichaeli* Debeaux. (10 g, Ranunculaceae, Aconiti lateralis radix praeparaia), and *Cinnamomum cassia* Presl. (10 g, Lauraceae, Cinnamomi cortex). The herbs were purchased from Beijing Tongrentang Co., Ltd. and identified by professor Jun He, Department of Pharmacy, China–Japan Friendship Hospital. Because of the poor solubility, XFP was made into water extract to reach the gavage dose. The preparation process of XFP decoction was as follows: The medicinal materials were mixed and made into powder, and then the powder was soaked in water at a ratio of 1:10 (v/v) for 1 h, decocted twice for 1 h each time after boiling. Finally, the collected liquid was concentrated into 100% decoction by rotary evaporation and stored at 4°C. The chemical fingerprints and ingredients of *Rheum officinale* Baill. and the XFP decoction were assessed by ultrahigh-performance liquid chromatography -Q exactive hybrid quadrupole -Orbit high resolution accurate mass spectrometry and presented in **Supplementary Material 1**.

### Induction of the SD-CIA Rat Modelsand Treatments

After 3 days of acclimation, the rats were randomly divided into five groups with 10 rats in each group: the control group, SD group, CIA group, SD-CIA group, and XFP-treated SD-CIA group. Among them, the SD rat model and SD-CIA rat model were induced as previously described (Xiao et al., 2007). 1) SD rat model: Rats were administered a dose of *Rheum officinale* Baill. decoction at 10 g/kg/day irregular eating to cause SD in the first 2 weeks. Then, the rats were administered a dose of *Rheum officinale* Baill. decoction at 5 g/kg/day and remained irregular eating from the third week to the end of the experiment. 2). CIA rat model: Rats were immunized by intradermal injection of 50 µg of bovine type II collagen (Chondrex, Inc., Redmond, WA, USA) emulsified in isopyknic incomplete Freund’s adjuvant (IFA) (Chondrex) at the base of the tail. Seven days later, the same preparation was given on the other side of the tail for booster immunization. 3). SD-CIA rat model: This model is a combination of the SD rat model and CIA rat model. Briefly, rats were administered *Rheum officinale* Baill. decoction and irregular eating to establish an SD rat model. After 2 weeks, the rats were administered the first immunization to establish the CIA rat model.

**Figure 1:**
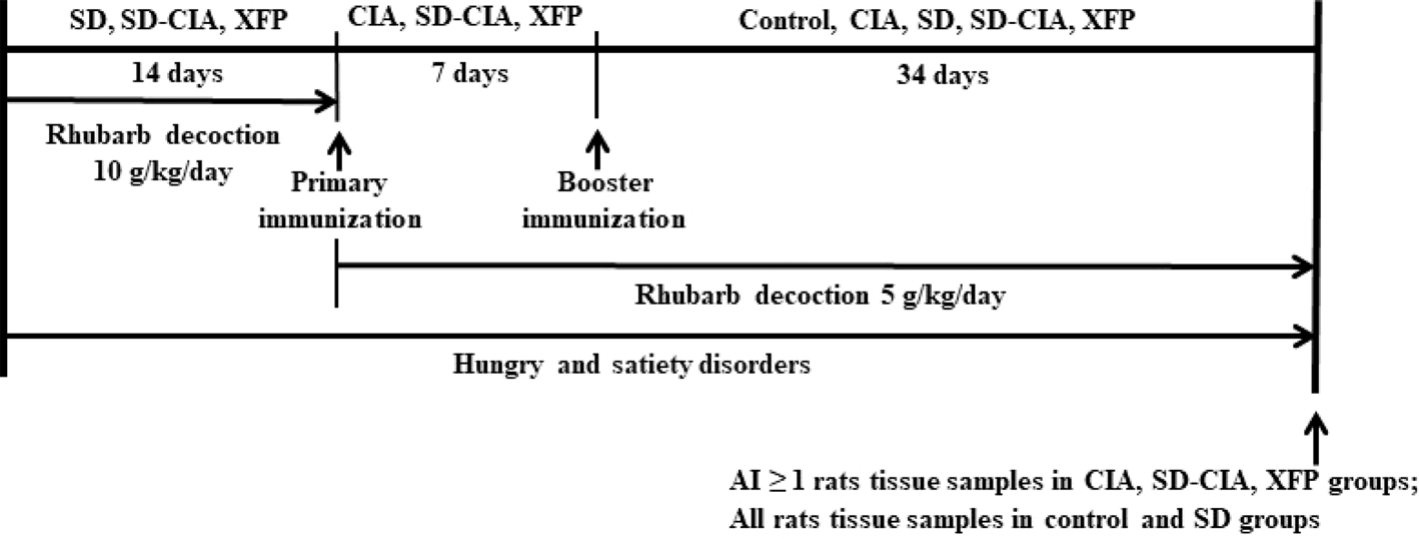
Experimental schedule. Male Sprague–Dawley rats were divided into the control, CIA, SD, SD-CIA, and XFP groups. The SD, SD-CIA, and XFP groups were administered *Rheum officinale* Baill. decoction at 10 g/kg/day in the first 2 weeks and at 5 g/kg/day until being sacrificed. In addition, these three groups were treated with irregular eating from the beginning to the end of this experiment. Next, the CIA, SD-CIA, and XFP groups were immunized with an intradermal injection of 50 µg of IFA-emulsified bovine type II collagen on the base of the tail twice at 7-day intervals. Control group and SD group were given the same amount of normal saline by the same method. Then, the control, CIA, SD, SD-CIA, and XFP groups were intragastrically administered treatment for 34 days. At the end of the experiment, tissue samples were collected from control and SD group rats with an AI ≥ 1 in the other groups. CIA, collagen-induced arthritis; SD, spleen deficiency; SD-CIA, collagen-induced arthritis with spleen deficiency; XFP, Xiong Fu powder; AI, arthritis index; IFA, incomplete Freund’s adjuvant.

After booster immunization in CIA and SD-CIA models, therapeutic intervention in rats began. The rats in the XFP group were administered XFP decoction at 23 g/kg/day (twice the human equivalent dose) once a day for 34 days. The other groups were intragastrically administered an equal volume of pure water (10 ml/kg/day). The entire experimental process is shown in **Figure 1**.

### Evaluation of SD Syndrome and Arthritis of the Hind Limbs

According to the classical theory of TCM, research on standardization of TCM syndromes and the biological characteristics of rats (Du et al., 2012), the state of SD model rats primarily includes listlessness, narrow eyes, dry and dull hair, hunched posture, reduced physical activity, huddled posture, reduced food intake, weight loss, and loose stools.

The extent of hind limb swelling was assessed every 3 days from the day of administration. According to conventional criteria (Xiao et al., 2006), the severity of arthritis in each hind leg was expressed by the arthritis index (AI) scores, which ranged from 0 to 4 points: 0, no swelling or oedema; 1, detectable erythema and oedema limited to the digits; 2, slight erythema and oedema from digits to tarsal bone; 3, moderate erythema and oedema from foot to ankle; 4, maximal erythema and oedema at entire ankle. The maximum AI score was 8 points (4 points × 2 hind legs).

### Microcomputed Tomography Analysis

Bone erosion was evaluated by microcomputed tomography (micro-CT). After 34 days of drug treatment, the left ankles and claws were collected and fixed in formalin for 7 days. Then, the samples were scanned using a SkyScan 1174 micro-CT scanner (Bruker, Belgium) to evaluate bone damage. N-Recon software was used for three-dimensional (3D) image reconstruction, and matched CT-AN software was used to quantify 3D images. The bone volume (BV) and bone surface (BS) of the tarsus were then calculated to assess changes in bone structure, and the BS/BV ratio was used to evaluate surface density.

### Evaluation of Histopathological and Tartrate-Resistant Acid Phosphatase Staining of Ankle Joints

Haematoxylin-eosin (H&E) staining and tartrate-resistant acid phosphatase (TRACP) staining were used for histological scoring and bone damage evaluation of the ankle joints. After micro-CT, the claws and ankles were decalcified in 10% ethylene diamine tetraacetic acid (EDTA)–Na_2_ for 1 month. After progressive dehydration and transparency, the samples were embedded in paraffin, prepared into 7-µm-thick slices, and finally stained with H&E. Histological scores were assessed by the destruction of cartilage damage and bone damage, cell infiltration, synovial hyperplasia, pannus respectively, and the mean value of the above indicators as the overall observation index, and were scored on a criterion of 0-3 (0, normal; 1, weak; 2, moderate; and 3, severe) (Li et al., 2020). TRACP (Sigma, St. Louis, MO, USA) staining assay was used to quantify the OCs of ankle joint slice samples, which were identified by the presence of TRACP-positive multinucleated cells that contained at least three nuclei. Typical images were captured using the Leica Qwin image analysis software (Leica Microsystem, Germany).

### Immunohistochemistry

The levels of sIgA in the jejunum and ileum were determined by immunohistochemistry (IHC) assay. After dewaxing and hydration, paraffin-embedded slices (5-µm-thick) of the jejunum and ileum were repaired with antigen by high pressure in citrate buffer (pH = 6.0). Then, the sample was incubated in 3% hydrogen peroxide (H_2_O_2_) in darkness, followed by goat serum blocking buffer at room temperature. Afterward, the ileum was incubated with goat anti-rat sIgA (Abcam, Cambridge, MA, USA; 1:400) overnight at 4°C and then treated with horseradish peroxidase–related species-specific secondary antibody for 20 min. The sections were stained with 3,3′-diaminobenzidine (DAB) and then counterstained with haematoxylin. Finally, random images were captured using a ZEISS Axio Observer 3 microscope, and the optical density of the positively stained area was examined using Image Pro-Plus.

### Enzyme-Linked Immunosorbent Assay

The levels of anti–collagen type II antibody (Col II Ab) in the serum and sIgA in the ileum were detected using enzyme-linked immunosorbent assay (ELISA). The blood, jejunum and ileum were collected after 34 days of treatment. After being obtained from the abdominal aorta, blood was centrifuged (3,000 rpm, 10 min, 4°C). The jejunum and ileum were homogenized in normal saline at a ratio of 1:10 (g/v) followed by centrifugation (3,000 rpm, 10 min, 4°C). Both serum and homogenate were collected and stored at −80°C. Col II Ab and sIgA were measured using ELISA kits following the manufacturer’s protocol. The sIgA ELISA kit was obtained from the Nanjing Jiancheng Bioengineering Institute (Nanjing, China). The Col II Ab ELISA kit was purchased from the Beijing Qisong Biotechnology Company (Beijing, China).

### Flow Cytometry

PPs in small intestines were isolated. Then, similar to our previous study (Li et al., 2020), Tregs, Th17 cells, and the Tregs/Th17 cells ratio in PPs were evaluated using a FACS Canto II flow cytometer (FACS Canto II, Becton, Dickinson, Co., Franklin Lakes, NJ, USA). Briefly, PPs from the five groups were ground into single cells and filtered through a cell sieve. Antibodies against CD4^+^ and IL-17^+^ were used to identify Th17 cells. Antibodies against CD4^+^, CD25^+^, and Foxp3^+^ were used to stain Tregs (BD Pharmingen, San Diego, CA, USA).

### Quantitative Real-Time Polymerase Chain Reaction

The quantitative real-time polymerase chain reaction (qRT-PCR) assay was used to detect the mRNA expression levels of tumor necrosis factor–α *(TNF-α), IL-1β, IL-6, IL-10*, and *IL-17* in the ileum. According to the manufacturer’s recommended procedure, total RNA of the sample in rats was extracted using TRIzol reagent (Invitrogen, Carlsbad, CA, USA). Then, the PrimeScript RT Reagent Kit with gDNA Eraser (TaKaRa, Tokyo, Japan) was used to conduct gDNA elimination and cDNA synthesis. SYBR premix Ex Taq (Tli RNaseH Plus) (TaKaRa) was used to perform qRT-PCR *via* a Quant Studio 5 real-time PCR system (Thermo Fisher Scientific, Waltham, MA, USA). The program was performed as follows: initial denaturation at 95°C for 30 s and amplification for 40 cycles at 95°C for 5 s and 60°C for 34 s. All experiments were repeated three times with three technical duplications, and the expression levels of relative mRNA were normalized to *β-actin* and determined using the 2^−△△Ct^ method. The primers used in this experiment are shown in **Supplementary Material 2**.

### Metagenomic Sequencing of the Gut Microbiota

The ileum intestinal contents were harvested, and microbial DNA was extracted using the Power Faecal DNA Kit (Qiagen, Valencia, CA, USA). After a series of pretreatments, the samples were amplified by PCR to form sequencing libraries. Then, the qualified libraries were sequenced using the Illumina sequencing platform. The original reads obtained by sequencing were quality-controlled and filtered to obtain clean reads. After splicing and assembly, the coding genes were predicted and annotated in the database. At the same time, clean reads were taxonomically analyzed, and the species composition, abundance of samples, and function based on the Kyoto Encyclopedia of Genes and Genomes (KEGG) database were statistically analyzed. In addition, association of pathways with dominant functions and the top 10 species in the XFP group was analyzed.

### Statistical Analysis

All data are shown as the mean ± *SEM*. One-way analysis of variance followed by the least significant difference test was used to determine differences between groups. The Kruskal–Wallis H and Mann–Whitney U tests were used when data were not normally distributed. Significance was defined as *P <* 0.05 using SPSS 20.0 software.

## Results

### XFP Ameliorates Arthritis Progression in SD-CIA

**Figure 2:**
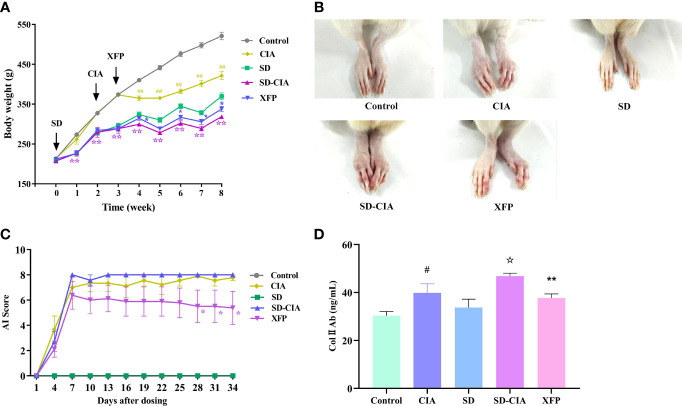
Therapeutic effect of XFP on a rat model of SD-CIA. **(A)** Body weight of each group. Weight was measured once a week. **(B)** Representative gross images of the ankle joint of each group on day 34 after treatment. **(C)** The line graph of AI scores every 3 days. **(D)** The levels of Col II Ab in the serum of each group. *n* = 10 (control and SD), *n* = 8 (CIA and SD-CIA), *n* = 9 (XFP)8. Data are presented as the mean ± *SEM*. *^#^P* < 0.05 and *^##^P* < 0.01 compared to the control group; *^☆^P* < 0.05 and *^☆☆^P* < 0.01 compared to the CIA group; ^*^P < 0.05 and ^**^P < 0.01 compared to the SD-CIA group. XFP, Xiong Fu powder; Col II Ab, anti–collagen type II antibody; SD-CIA, collagen-induced arthritis with spleen deficiency; AI, arthritis index; CIA, collagen-induced arthritis; SD, spleen deficiency.

After 34 days of XFP treatment, the symptoms of SD and arthritis progression in SD-CIA were ameliorated. Rats in the SD and SD-CIA groups were listless with dull hair and loose stools. After treatment with XFP, the state of the rats was improved, their hair became glossy, and their stool was molded. Similarly, as another important index for evaluating SD, as shown in **Figure 2A**, the body weight of rats was much lower in the SD and SD-CIA groups than in the control group from the first week of *Rheum officinale* Baill. intervention (*P* < 0.01), whereas the body weight was increased in the XFP group after 1 week of treatment compared with the SD-CIA group (*P* < 0.05). These results indicated that XFP ameliorated arthritis with SD.

To determine the therapeutic effect of XFP in arthritis, the AI scores at the lesion location and serum Col II Ab in entirety were used. At the end of the experiment, more severe hind limb swelling occurred in the SD-CIA group than in the CIA group, and this swelling was distinctly relieved in the XFP group compared to the SD-CIA group (**Figure 2B**). The AI scores are shown in **Figure 2C**. Arthritis in the control and SD groups was as expected, with an AI score of 0. Seven days after modeling, the AI scores of the CIA model had nearly peaked. In general, the SD-CIA group displayed higher AI scores than the CIA group. After treatment, from day 28 onward, AI scores in the XFP group were significantly lower than those in the SD-CIA group (*P* < 0.05). The levels of Col II Ab in serum exhibited a similar trend to AI scores (**Figure 2D**). Col II Ab levels were significantly increased in the CIA group compared with the control group (*P* < 0.05), and Col II Ab levels in the SD-CIA group were significantly different from those in the CIA group (*P* < 0.05). After XFP treatment, the serum levels of Col II Ab were significantly lower than those of the SD-CIA group (*P* < 0.01) (**Figure 2D).** These findings suggested that although SD did not directly cause the formation of CIA, it significantly aggravated CIA. Furthermore, XFP showed the potential to slow the progression of arthritis with SD.

### XFP Alleviates the Severity of Bone Erosion and Bone Destruction in SD-CIA

**Figure 3:**
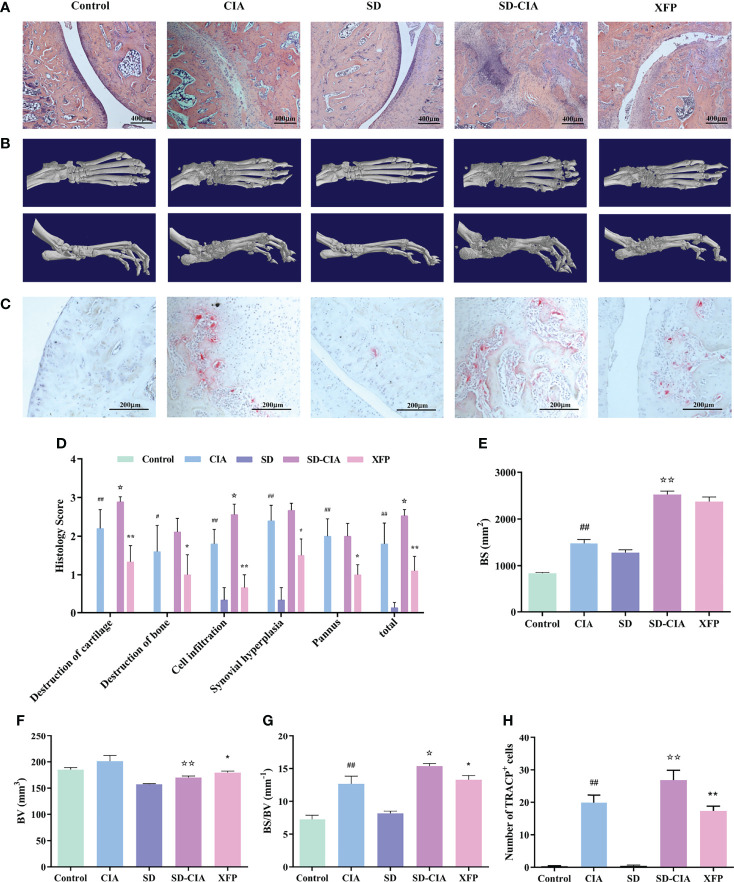
Reduced bone destruction and joint damage effects of XFP in SD-CIA. **(A)** Representative images of ankle joint pathology shown through H&E staining. **(B)** Representative images of ankle joint micro-CT after treatment. **(C)** OCs in ankle was performed via TRACP staining. **(D)** Histological score of the ankle joints in each group shown by H&E staining. **(E–G)** Line plots showing the BS, BV, and BS/BV values of all groups. **(H)** OC counts after staining. The TRACP-positive multinucleated cells (>3 nuclei) were enumerated. *n* = 8. Data are presented as the mean ± *SEM*. *^#^P* < 0.05 and *^##^P* < 0.01 compared to the control group; *^☆^P* < 0.05 and *^☆☆^P* < 0.01 compared to the CIA group; ^*^P < 0.05 and ^**^P < 0.01 compared to the SD-CIA group. XFP, Xiong Fu powder; CIA, collagen-induced arthritis; SD, spleen deficiency; SD-CIA, collagen-induced arthritis with spleen deficiency; micro-CT, microcomputed tomography; H&E, haematoxylin-eosin; OCs, osteoclasts; BS, bone surface; BV, bone volume.

Next, changes in ankle joint pathology were examined using H&E staining (**Figures 3A, D** **)**. Compared with the control group, the conditions of cartilage destruction, bone destruction, cell infiltration, synovial hyperplasiaand pannus formation were significantly aggravated in CIA group (P < 0.05, P < 0.01); Compared with CIA group, the above indexes were heavier (P < 0.05); Compared with SD-CIA group, XFP group showed significant improvement in these indicators (P < 0.05, P < 0.01).Taking the mean value of the above indicators as the overall observation index, the trend is similar to the above trend.

In addition, micro-CT was used to evaluate the therapeutic effect of XFP on bone injury, and 3D reconstruction of the ankle joint is shown in **Figure 3B**. The BS, BV, and BS/BV values of the inflamed ankle were measured to quantify the degree of bone remodeling in the different groups (**Figures 3E–G** **)**. The results demonstrated that the BS value of CIA was significantly different from that of the control group (*P* < 0.01), and the BS value in the SD-CIA group was higher than that in the CIA group (*P* < 0.01). After XFP treatment, the BS value had a decreasing tendency, but there was no significant difference compared to SD-CIA. The BV value was decreased in the SD-CIA group compared to the CIA group (*P* < 0.01), and XFP treatment increased it compared to the SD-CIA group (*P <* 0.05). With respect to the BS/BV value, we found that the CIA group still had bone destruction compared to the control group (*P* < 0.01), and the tendency of BS/BV was higher in the SD-CIA group than in the CIA group (*P <* 0.05). After XFP treatment, the BS/BV value was lower than that in the SD-CIA group (*P* < 0.05). On the one hand, the micro-CT results preliminarily confirmed our prediction that SD could aggravate the development of arthritis. These results also suggested that XFP inhibited joint damage.

Furthermore, the number of OCs in the ankle joints was measured using the TRACP assay to determine the severity of bone destruction. The results of TRACP (**Figures 3C, H**) showed that the number of OCs in the CIA group increased significantly compared to that in the control group (*P* < 0.01), and there was a significant difference between the SD-CIA and CIA groups (*P* < 0.01). In response to XFP intervention, the number of OCs in the XFP group decreased significantly compared to that in the SD-CIA group, which was also statistically significant (*P* < 0.01). The experiment also demonstrated that SD aggravated the bone destruction of joints, and XFP exerted a good therapeutic effect on SD-CIA.

In conclusion, SD might aggravate the development of CIA, whereas XFP exerted an obvious therapeutic benefit on SD-CIA.

### XFP Regulates the Immune Status of Intestinal PPs

**Figure 4:**
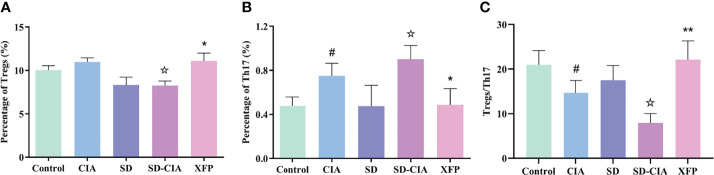
The modulatory effect of XFP on the immune status of the PPs. The percentages of Tregs and Th17 cells and the Tregs/Th17 ratio in PPs were detected by flow cytometry. **(A)** Bar charts showing the percentage of Tregs by flow cytometry. **(B)** Bar charts showing the percentage of Th17 cells by flow cytometry. **(C)** The ratio of Tregs/Th17 cells in each group. *n* = 8. Data are presented as the mean ± *SEM*. *^#^P* < 0.05 compared to the control group; *^☆^P* < 0.05 compared to the CIA group; ^*^P < 0.05 and ^**^P < 0.01 compared to the SD-CIA group. XFP, Xiong Fu powder; CIA, collagen-induced arthritis; SD, spleen deficiency; SD-CIA, collagen-induced arthritis with spleen deficiency; PPs, Peyer’s patches; Tregs, T regulatory cells; Th17, T helper 17.

To investigate the effect of XFP on the PPs of the intestine, the percentages of Tregs and Th17 cells and the ratio of Tregs/Th17 cells in the PPs were evaluated using flow cytometry. The percentage of Tregs in the SD-CIA group was lower than that in the CIA group (*P* < 0.05). In contrast, the Treg percentage was higher in the XFP group than in the SD-CIA group (*P* < 0.05) (**Figure 4A**). The proportion of Th17 cells in the CIA group was upregulated compared to that in the control group (*P* < 0.05), and the SD-CIA group displayed a higher tendency than the CIA group (*P* < 0.05). However, after treatment with XFP, the proportion of Th17 cells was decreased compared to that in the SD-CIA group (*P* < 0.05) (**Figure 4B**). The ratio of Tregs/Th17 cells was decreased in the CIA group compared with the control group (*P* < 0.05), and in the SD-CIA rats, there was a lower tendency compared to the CIA group (*P* < 0.05). After XFP treatment, the Tregs/Th17 cells ratio was significantly upregulated compared to that in the SD-CIA group (*P* < 0.01) (**Figure 4C**). These results demonstrated that SD had a detrimental effect on immune function and that XFP treatment exerted an extensive regulatory effect on intestinal mucosal immune function in SD-CIA rats.

### XFP Modulates the Immune Status of the Ileum

**Figure 5:**
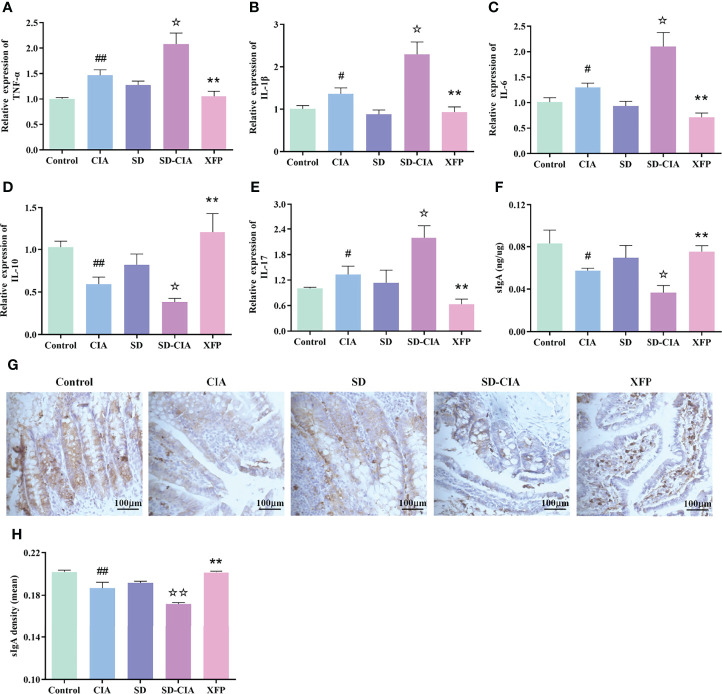
The regulatory effect of XFP on immune status of jejunum and ileum. **(A–E)** RNA expression of *TNF-α, IL-1β, IL-6, IL-10*, and in the jejunum and ileum. **(F)** The relative concentration of sIgA in the jejunum and ileum as measured by ELISA. **(G)** Representative images of the expression of sIgA in the jejunum and ileum obtained by IHC. **(H)** Relative expression of sIgA in the jejunum and ileum via IHC. *n = 8*. Data are presented as the mean ± SEM. *^#^P* < 0.05 and *^##^P* < 0.01 compared to the control group; *^☆^P* < 0.05 and *^☆☆^P* < 0.01 compared to the CIA group; ^**^P < 0.01 compared to the SD-CIA group. XFP, Xiong Fu powder; CIA, collagen-induced arthritis; SD, spleen deficiency; SD-CIA, collagen-induced arthritis with spleen deficiency; TNF, tumor necrosis factor; IL, interleukin; qRT-PCR, quantitative real-time polymerase chain reaction; sIgA, secretory immunoglobulin A; ELISA, enzyme-linked immunosorbent assay; IHC, immunohistochemistry.

The influence of XFP on ileal immune status was evaluated by detecting the expression of genes in the ileum using a qRT-PCR assay (**Figures 5A–E**). The CIA group exhibited upregulated expression of *TNF-α, IL-1β, IL-6,* and *IL-17* and downregulated *IL-10* levels in the ileum compared with the control group (*P* < 0.05 and *P* < 0.01, respectively), and the trend in the SD-CIA group was more significant than that in the CIA group (*P* < 0.05). Intervention with XFP significantly decreased *TNF-α, IL-1β, IL-6,* and *IL-17* levels and increased levels of *IL-10* compared to those in the SD-CIA group (*P* < 0.01) (**Figures 5A–E**). These results suggested that although SD had no significant effect on the immune state of the ileum, it aggravated the immune state disorder in CIA rats, and XFP treatment had an obvious therapeutic effect on the immune state of SD-CIA.

Because of the obvious variation in the contents of the intestinal tract, it is speculated that these effects may be caused by metabolites or antigens related to the intestinal microbiota. Therefore, we also examined the expression of sIgA using ELISA and IHC because sIgA is known to regulate intestinal microorganisms. ELISA suggested that sIgA levels in the CIA group were significantly lower than those in the control group (*P* < 0.05) and that sIgA levels in the SD-CIA group were lower than those in the CIA group (*P* < 0.05). XFP treatment upregulated sIgA levels compared to the SD-CIA group (*P* < 0.01) (**Figure 5F**). IHC experiments revealed that the protein expression density of sIgA in the CIA group was significantly decreased compared to that in the control group (*P* < 0.01). The density in the SD-CIA group was also significantly lower than that in the CIA group (*P* < 0.01). After XFP intervention, the density of sIgA protein expression increased significantly compared with that in the SD-CIA group (*P* < 0.01) (**Figures 5G, H**). These results suggested that CIA affected the intestinal tract mucosal immune barrier to a certain extent and reduced the pathogenic microorganism’s clearance rate, and the SD model might exacerbate this effect. In addition, XFP treatment affected sIgA levels, indicating that XFP treatments reshaped the immune barrier and reversed the regulatory function of the intestinal tract on intestinal bacteria.

The above results indicated that the immune barrier in CIA was impaired, and SD further aggravated the immune disorder in CIA, leading to a reduced clearance rate of pathogenic microorganisms and aggravating the occurrence of CIA. XFP treatment improved immune status and alleviated SD-CIA.

### XFP Alters the Distribution of the Gut Microbiota in the Ileum

**Figure 6:**
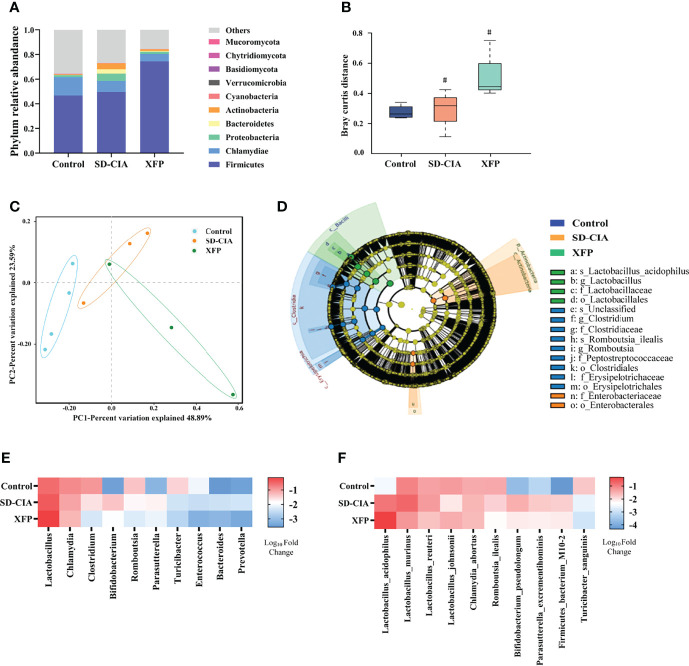
Effect of XFP on gut microbiota distribution in the ileum. **(A)** Relative abundance of the top 10 most abundant phyla in each group. **(B)** Boxplot of species composition based on Anosim. Data are presented as the medians (IQRs). **(C)** Differences in gut microbial communities among the groups as determined by PCoA. **(D)** The evolutionary branching diagram of gut microbiota using LEfSe analyses. Relative abundances among the groups at the genus **(E)** and species **(F)** levels. The data were transformed by log_(10)_. N = 4 (control), N = 3 (SD-CIA and XFP). ^#^ *P* < 0.05 compared to the control group. XFP, Xiong Fu powder; CIA, collagen-induced arthritis; SD, spleen deficiency; SD-CIA, collagen-induced arthritis with spleen deficiency; IQR, interquartile range; Anosim, analysis of similarities; PCoA, principal coordinates analysis; LEfSe, linear discriminant analysis effect size.

An increasing number of studies have shown that intestinal microorganisms play an important role in intestinal immune regulation (Xu C et al., 2020; Xu Z et al., 2020). Therefore, metagenomic sequencing of ileum contents was used to detect changes in the gut microbiota (**Figure 6**). Among the top 10 phyla in terms of abundance, *Firmicutes* were dominant, followed by *Chlamydiae.* The abundance of *Firmicutes* in the XFP group and *Chlamydiae* in the control group and *Proteobacteria*, *Bacteroidetes*, and *Actinobacteria* in the SD-CIA group was highest among the three groups. In addition, other phyla accounted for a certain proportion (**Figure 6A**). Next, the analysis of similarities (Anosim) was used to assess bacterial community similarity. The results showed that compared with the control group, the SD-CIA group and the XFP group were significantly different in their distribution of microflora at the genus level. However, there were no differences between the SD-CIA group and the XFP group (**Figure 6B**). The distribution of samples in each group was relatively concentrated, but one sample in the XFP group overlapped with the region in the SD-CIA group as determined using principal coordinates analysis (PCoA) (**Figure 6C**). Furthermore, linear discriminant analysis effect size (LEfSe) was used to identify the biomarkers in each group (**Figure 6D**). The control group had the most types of biomarkers, whereas *L. acidophilus* and its different biological classifications dominated in the XFP group. Finally, the 10 genera and species with the highest abundance in each group are shown in **Figures 6E,F**. Consistent with LEfSe, the abundance of *Lactobacillus* and *L. acidophilus* was highest in the XFP group. Specifically, at the species level, the abundances of *L. murinus*, *L. reuteri*, *B. pseudolongum*, *P. excrementihominis*, and *F. bacterium M10-2* in the SD-CIA group were upregulated compared with those in the control group. Compared with the SD-CIA group, the abundance of the above bacteria was decreased in the XFP group. However, *L. johnsonii* exhibited the opposite trend. These results illustrated that SD-CIA altered the distribution of bacterial flora and that XFP restored bacterial community disorder to a certain extent.

In addition, according to the literature, we found that among the 10 strains with the highest abundance, five strains, including *L. acidophilus* (Yanagihara et al., 2014; Chen et al., 2015; Li et al., 2019; Pan et al., 2019), *L. murinus* (Wilck et al., 2017; Bernard-Raichon et al., 2021), *L. johnsonii* (Lau et al., 2011; Zheng et al., 2021), *L. reuteri* (Mu et al., 2017), and *B. seudolongum* (Bromberg et al., 2020), showed the ability to regulate Tregs and Th17 cells. This suggested that the therapeutic effect of XFP on SD-CIA rats might be related to the regulation of the intestinal microenvironment in which bacteria participate.

### XFP Alters the Function of the Gut Microbiota in the Ileum

**Figure 7:**
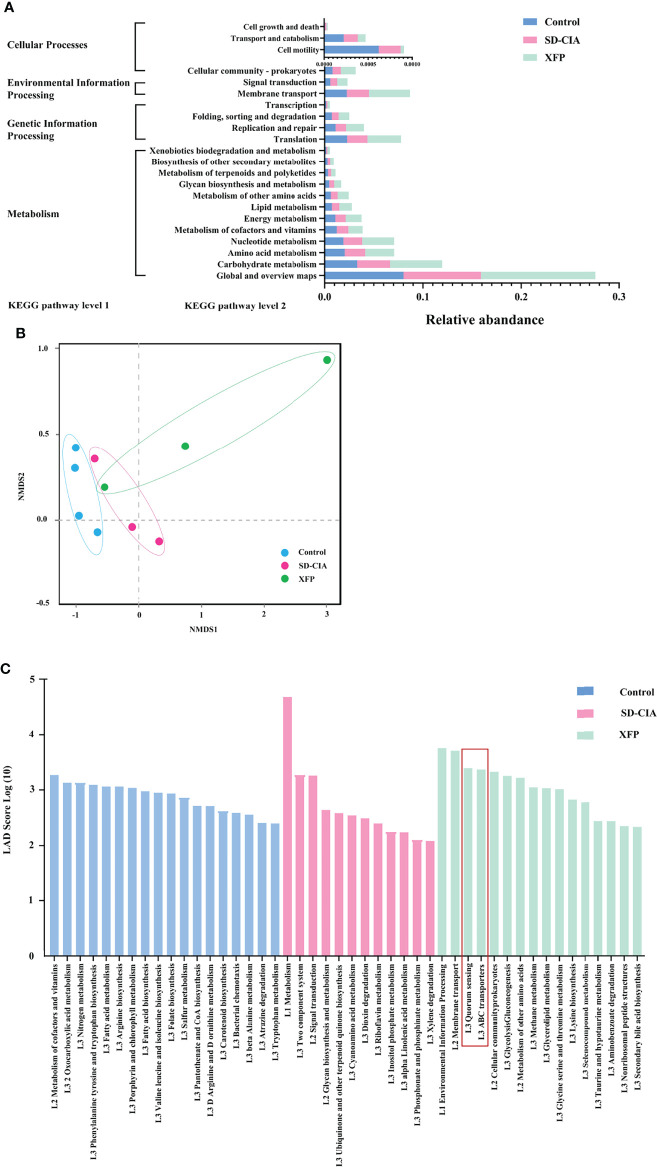
Effect of XFP on the function of gut microbiota in ileum contents. **(A)** Statistical map of KEGG metabolic pathway-related functional genes at level 2 in each group. **(B)** The differences in gut microbial communities among the groups by NMDS. **(C)** Diagram of the function of gut microbiota using LEfSe analyses. The data were transformed by log_(10)_. *n* = 4 (control), n = 3 (SD-CIA and XFP). XFP, Xiong Fu powder; SD-CIA, collagen-induced arthritis with spleen deficiency; NMDS, non-metric multidimensional scaling; LEfSe, linear discriminant analysis effect size; LDA, linear discriminant analysis.

Furthermore, we conducted a comprehensive analysis of the functional modules in the gut microbiome to explore the potential mechanism underlying the effects of XFP administration using the KEGG database. At KEGG pathway level 1, Metabolism, Genetic Information Processing, and Cellular Processes pathways were identified. At KEGG pathway level 2, 22 pathways were enriched, including pathways related to the maintenance of bacterial survival: carbohydrate metabolism, amino acid metabolism, nucleotide metabolism, and energy metabolism under Metabolism of KEGG pathway level 1; the gene-related pathways: translation, replication, and repair under Genetic Information Processing of KEGG pathway level 1; and the pathways including signal transduction and cell motility belonging to Environmental Information Processing and Cellular Processes in level 1 (**Figure 7A**). The abundance of pathways generally conforms to conventional understanding. Then, sample similarity was assessed using non-metric multidimensional scaling (NMDS) analysis (**Figure 7B**). Except for one sample in the XFP group, the sample distribution of each group was relatively concentrated, suggesting that the composition of functional genes was similar within the group and different among the groups. Furthermore, LEfSe was used to identify pathways with significant differences among the groups. Several dominant pathways were obtained in each group. As the smallest unit of the pathway, quorum sensing contributed the most to KEGG pathway level 3 in the XFP group, followed by ABC transporters (**Figure 7C**).

Finally, we analyzed the association between the intestinal bacteria with high abundance and the KEGG pathway in level 3 that exerted a functional advantage in the XFP group. The results showed that except for *C. abortus*, *F. bacterium M10-2*, and *P. excrementihominis*, the other seven species were associated with the dominant pathways in the top 10 most abundant species. The pathways related to the high abundance of strains in the XFP group were quorum sensing and ABC transporters (**Table 1**). Recent reports have shown that the quorum sensing mechanism increases adherence to intestinal epithelial cells and that *L. acidophilus* can adhere to the intestinal epithelium and promote communication with the immune system (Buck et al., 2009). In addition, research has found that other strains can modulate immune responses *via* quorum sensing (Kariminik et al., 2017). Other pathways, such as those involving ABC transporters, are key processes to achieve self-immunity of bacteria (Smits et al., 2020).

**Table 1:** Association analysis of pathways with dominant functions and the top 10 species in the XFP group.

Taxonomy	Pathway ID	Pathway Name	KO	KO function
*L. acidophilus*	ko02024	Quorum sensing	K03217	YidC/Oxa1 family membrane protein insertase
			K03076	Preprotein translocase subunit SecY
	ko02010	ABC transporters	K05847	Osmoprotectant transport system ATP-binding protein
			K16785	Energy-coupling factor transport system permease protein
*L. murinus*	ko02024	Quorum sensing	K03076	Preprotein translocase subunit SecY
	ko02010	ABC transporters	K05847	Osmoprotectant transport system ATP-binding protein
			K16785	Energy-coupling factor transport system permease protein
*L. johnsonii*	ko02024	Quorum sensing	K03217	YidC/Oxa1 family membrane protein insertase
			K03076	Preprotein translocase subunit SecY
	ko02010	ABC transporters	K05847	Osmoprotectant transport system ATP-binding protein
			K16785	Energy-coupling factor transport system permease protein
*L. reuteri*	ko02024	Quorum sensing	K03217	YidC/Oxa1 family membrane protein insertase
			K16785	Energy-coupling factor transport system permease protein
*B. pseudolongum*	ko02010	ABC transporters	K16785	Energy-coupling factor transport system permease protein
			K17318	Putative aldouronate transport system substrate-binding protein
			K17320	Putative aldouronate transport system permease protein
*R. ilealis*	ko02024	Quorum sensing	K03217	YidC/Oxa1 family membrane protein insertase
			K03076	Preprotein translocase subunit SecY
	ko02010	ABC transporters	K05847	Osmoprotectant transport system ATP-binding protein
			K16785	Energy-coupling factor transport system permease protein
			K17318	Putative aldouronate transport system substrate-binding protein
			K17320	Putative aldouronate transport system permease protein
*T. sanguinis*	ko02024	Quorum sensing	K03217	YidC/Oxa1 family membrane protein insertase
	ko02010	ABC transporters	K16785	Energy-coupling factor transport system permease protein

## Discussion

In the theory of TCM, “spleen” is a comprehensive functional unit involved in digestion and absorption, energy conversion, immunity, and the neuroendocrine system, and the syndrome of SD is a comprehensive manifestation of the reduced functions of these systems. In particular, clinical and experimental studies have shown that SD syndrome has a close relationship with immunology. Low immune function makes the body more susceptible to diseases and less likely to recover. Our available data indicated that SD-CIA rats displayed more severe joint inflammation and bone destruction than CIA rats, suggesting that SD aggravates the severity of CIA. XFP is an effective prescription for treating SD-CIA under the guidance of TCM theory (Xiao et al., 2007). Our results showed that XFP significantly alleviated SD-CIA joint swelling, inflammation, and joint bone destruction, suggesting that XFP exerts a beneficial effect on SD-CIA.

The intestinal microenvironment is primarily composed of the intestinal mucosal immune system and the gut microbiota, and these two systems affect each other to jointly maintain homeostasis of the intestinal microenvironment (Catrina et al., 2016). In a CIA model of animals and patients with RA, the intestinal mucosal immune system and gut microbiota were abnormal to varying degrees (Lucchino et al., 2019; Kishikawa et al., 2020). In addition, our previous studies have demonstrated this pathological change (Li et al., 2020).

Th17 cells and Tregs are abundant within the intestinal mucosa, where they protect the host from pathogenic microorganisms and restrain excessive effector T-cell responses, respectively (Omenetti and Pizarro, 2015). Acting as a bridge between intestinal mucosal immunity and the gut microbiota, Tregs and Th17 cells can affect the gut microbiota by regulating the secretion of sIgA. On the other hand, bacteria and their metabolites can also regulate the generation of intestinal Tregs or expansion of Th17 cells, which can actively affect intestinal mucosal immunity (Omenetti and Pizarro, 2015). Our study found that the proportion of Tregs/Th17 cells of PPs in the SD-CIA group was reduced, and this proportion was restored after XFP intervention. After modeling, TNF-α, IL-1β, IL-6, and IL-17, which are associated with Th17 cells function, were significantly upregulated, and IL-10, which is associated with Tregs, was significantly downregulated. After XFP intervention, the immune status of these disorders was improved. Moreover, sIgA secretion was, as expected, significantly reduced in the SD-CIA group, which was reversed after XFP treatment. These results are similar to those of other studies (Tong et al., 2015) and suggest that an intestinal mucosal immune disorder with Tregs/Th17 cells as the core in SD-CIA, with XFP administration, improves this situation.

Increasing evidence indicates that disorders of the gut microbiota are involved in the pathogenesis of RA (Kishikawa et al., 2020), and the process of RA can be alleviated by regulating Tregs and Th17 cells in the intestinal mucosa to improve the gut microbiome. After administering *L. casei* to AIA rats, Pan et al. found that arthritis symptoms were relieved and that the associated gut microbiota, especially *Lactobacillus* and *L. acidophilus*, as well as immune disorders were regulated (Pan et al., 2019). Interestingly, our results also suggested significant enrichment of *Lactobacillus* and *L. acidophilus* in response to XFP treatment. Many studies have shown that species from *Lactobacillus*, such as *L. Acidophilus*, *L. Murinus*, *L. johnsonii*, and *L. reuteri*, which were enriched, can modulate host immune responses, especially by regulating Tregs and Th17 cells (Yanagihara et al., 2014; Mu et al., 2017; Bernard-Raichon et al., 2021; Zheng et al., 2021), and *L. johnsonii* was verified in a double-blind, randomized trial in healthy adults (Marcial et al., 2017). In addition, another bacterium with high abundance, *B. pseudolongum*, has also been shown to regulate Treg-related IL-10 (Bromberg et al., 2020). On the basis of the discussion above, XFP regulates mucosal immunity by increasing the abundance of Tregs and Th17-related probiotics, affecting CIA bone metabolism.

In addition to abundance, the function of intestinal flora in RA was also changed. Kishikawa et al. found that the abundance of R6FCZ7 (REDOX related) in the metagenome of patients with RA was significantly lower than that of healthy controls, whereas metabolism-related pathways (fatty acid biosynthesis, mucoglycan degradation, etc.) were significantly enriched (Kishikawa et al., 2020). Because of species differences, the pathways enriched in the SD-CIA group were metabolism-related pathways, such as cyanoamino acid metabolism. After treatment with XFP, quorum sensing and ABC transporters, etc., made high functional contributions. Bacterial quorum-sensing molecules are one of the primary means allowing communication between bacterial cells or populations. Recent reports have shown that the quorum sensing mechanism of *L. acidophilus* increases adherence to intestinal epithelial cells (Buck et al., 2009). In addition, quorum sensing modulates immune responses (Kariminik et al., 2017). ABC transporters have multiple roles, including nutrient uptake, exporting signaling molecules or toxic compounds such as xenobiotics and toxic metabolites, as well as conferring cells with multidrug resistance. Nutrient uptake is typically mediated by ABC importers, whereas ABC exporters mediate the export of toxic molecules out of cells, so this pathway is a key process for achieving self-immunity of bacteria (Smits et al., 2020). Therefore, he mechanism by which XFP improves SD-CIA may be related to quorum sensing increasing probiotic adhesion and maintaining autoimmunity through ABC transporters. However, the specific mechanism of XFP in increasing probiotic adhesion and maintaining the autoimmunity of strains needs to be further verified.

## Conclusions

**Figure 8:**
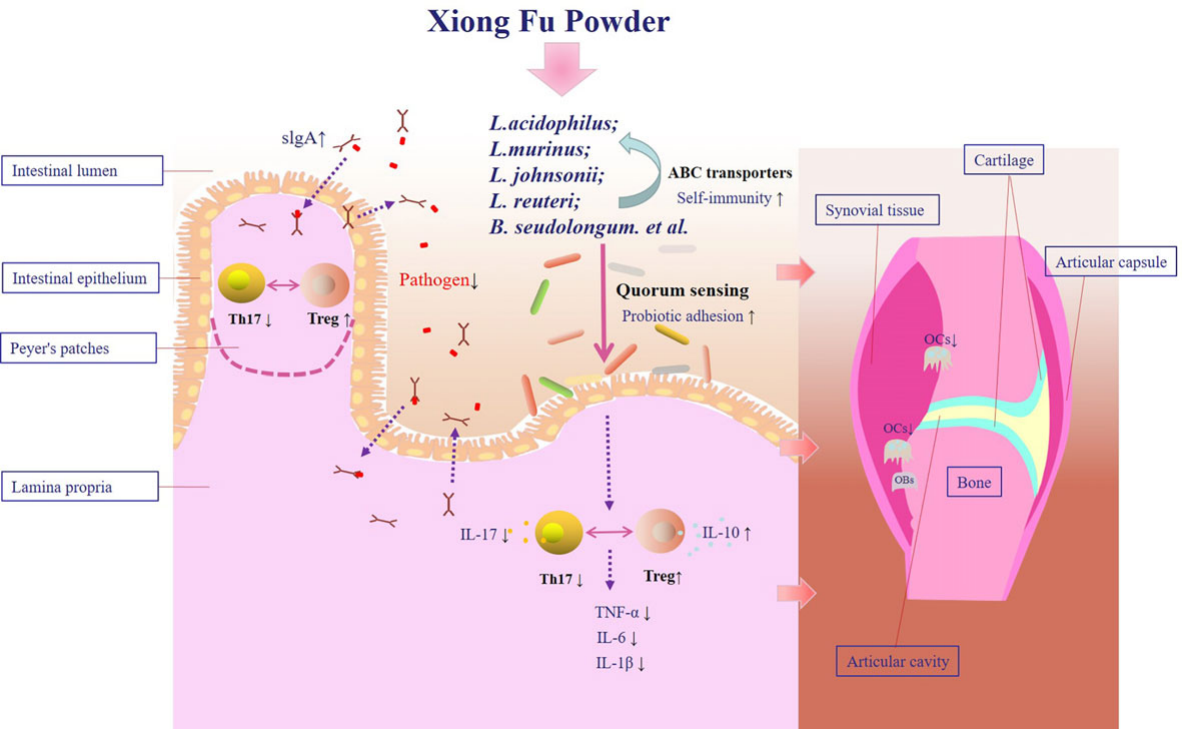
Therapeutic mechanism of XFP in the SD-CIA rat models. XFP, Xiong Fu powder; CIA, collagen-induced arthritis; SD, spleen deficiency; SD-CIA, collagen-induced arthritis with spleen deficiency.

SD can aggravate bone destruction in CIA. Compound XFP may attenuate SD-CIA bone destruction by regulating the intestinal microenvironment. One of the mechanisms is the cross-talk between sIgA secretion regulated by intestinal mucosal Tregs and Th17 cells and adhesion of *Lactobacillus* mediated by quorum sensing **(** **Figure 8** **)**.

## Data Availability Statement

The datasets presented in this study can be found in online repositories. The name of the repository and accession number can be found below: SRA, NCBI; PRJNA801062.

## Ethics Statement

All the experimental procedures were reviewed, approved, and directed by the Institute of Clinical Medical Science, China–Japan Friendship Hospital (Ethics No. 190104).

## Author Contributions

XYL, XX, and QY contributed equally to this study and share first authorship. CX formulated the concept and designed the study. XYL and XCL performed the experiments. YX, DF, and XYL analyzed the data.XYL, XX, and QY drafted the paper. CX, XX, and QY revised the paper. All authors contributed to the article and approved the submitted version.

## Funding

This study was financially supported by the National Natural Science Foundation of China (Grant number 81673844).

## Conflict of Interest

The authors declare that the research was conducted in the absence of any commercial or financial relationships that could be construed as a potential conflict of interest.

## Publisher’s Note

All claims expressed in this article are solely those of the authors and do not necessarily represent those of their affiliated organizations, or those of the publisher, the editors and the reviewers. Any product that may be evaluated in this article, or claim that may be made by its manufacturer, is not guaranteed or endorsed by the publisher.
