# Supplementary material for: Xiong Fu Powder Regulates the Intestinal Microenvironment to Protect Bones Against Destruction in Collagen-Induced Arthritis Rat Models
Source: Front Cell Infect Microbiol. 2022 Jul 1;12:854940. doi: 10.3389/fcimb.2022.854940 (PMC9285403; doi:10.3389/fcimb.2022.854940)
Supplement: Supplementary file 1 [file DataSheet_1.docx]

**Xiong Fu Powder regulates the intestinal microenvironment to protect bones against destruction in collagen-induced arthritis rat modelsXiaoya Li, Xiaoyu Xi, Qinbin Ye, Xiangchen Lu, Danping Fan, Ya Xia, Cheng Xiao**

Xiong Fu powder (XFP) is composed of the following herbs: *Astragalus mongholicus* Bunge. (15 g, Fabaceae, Astragali radix), *Atractylodes macrocephala* Koidz. (15 g, Asteraceae, Atractylodis macrocephalae rhizome), *Saposhnikovia divaricata* (Turcz. Ex Ledeb.) Schischk. (10 g, Apiaceae, Saposhnikoviae radix), *Glycyrrhiza glabra* L. (10 g, Fabaceae, Glycyrrhizae radix et rhizome), *Rehmannia glutinosa* (Gaertn.) DC. (15 g, Scrophulariaceae, Rehmanniae radix praeparata), *Angelica sinensis* (Oliv.) Diels. (10 g, Apiaceae, Angelicae sinensis radix), *Ligusticum chuanxiong* Hort. (10 g, Apiaceae, Chuanxiong rhizoma), *Bupleurum chinense* DC. (10 g, Apiaceae, Bupleuri radix), *Aconitum carmichaeli* Debeaux. (10 g, Ranunculaceae, Aconiti lateralis radix praeparaia), and *Cinnamomum cassia* Presl. (10 g, Lauraceae, Cinnamomi cortex). The herbs were purchased from Beijing Tongrentang Co., Ltd. and identified by professor Jun He, Department of Pharmacy, China-Japan Friendship Hospital. The composition of *Rheum officinale* Baill. and XFP is complex. A reliable quality extracts is the first step in a vivo trial. Therefore, the contents of decoction from *Rheum officinale* Baill. and XFP were analyzed by ultrahigh-performance liquid chromatography -Q exactive hybrid quadrupole -Orbit high resolution accurate mass spectrometry (UHPLC-Q-Orbitrap HRMS) to get chemical fingerprints. The detailed methods are illustrated in the following sections.

**1 Materials and methods**

**1.1 Sample preparation**

Decoctions from *Rheum officinale* Baill. and XFP (100 µL) were accurately transferred to a centrifuge tube, and 400 µL methanol (HPLC-grade, Aladdin, Shanghai, China) was added respectively. Then, the sample was swirled for 2 min to mix thoroughly and ultrasonicated for 40 min for full dissolution, followed by centrifuging (14000×g, 15 min, 4 °C). Finally, the supernatant was lyophilized and dissolved in 200 µL 20% methanol again. The supernatant was stored for later analysis.

**1.2 UHPLC**

UHPLC separation was performed by the UltiMate 3000 liquid chromatograph, which equipped with the quaternary pump, on-line degasser, thermostatic column chamber (Thermo Scientific, Sunnyvale, CA, USA) and the PAL autosampler (CTC Analytics, Zwingen, Switzerland's). A Waters ACQUITY BEH C8 (2.1 mm×100 mm, 1.7 μm) and a Waters ACQUITY HSS T3 analytical column (2.1 mm×100 mm, 1.8 μm) were used at a temperature of 50℃. Use an optimized gradient at the constant flow rate of 0.35 mL/min using 0.1% formic acid Milli-Q water (Solvent A) and acetonitrile (Solvent B) in the mode of positive ion, or using the 6.5 mM ammonium bicarbonate Milli-Q water (Solvent A) and 95% methanol (Solvent B) in the negative ion mode. The original conditions were set at 5% B, and kept for 1 min, and then programmed to 100% B at 24 min (in the positive ion mode) and held for 3.5 min or 18 min (in the negative ion mode) and held for 4 min. Finally, the gradient was restored to the original state at 27.6 min, and then was rebalanced for 2.5 min to complete the run in the positive ion mode. For the negative ion mode, the gradient was restored to the original conditions at 22.1 min and then rebalanced for 3min. Each injecting sample volume was 5 μL with 200 μL of 20% methanol aqueous solution needle washing solvent.

**1.3 Q-Orbitrap HRMS**

During the process, the UHPLC system was employed in conjunction with the benchtop Q-Orbitrap HRMS (Thermo Scientific, Bremen, Germany). The targets analyte ionization was performed by a heating electrospray ionization (HESI) source in ESI-mode. the voltage of spray is 3.8Kv/-3.0kV in the positive/negative ion mode. The corresponding parameters were as follows: the 320 ℃ capillary temperature; 350 ℃ auxiliary gas heater temperature; sheath gas, auxiliary gas and flow rate of 35 and 8 (in any unit); In the mass ranging of mass-to-charge ratio (M /z) 70-1050, Full ms resolution was70000, MS/MS resolution was 17500, TopN was 5 and NCE/stepped NCE: 20, 40.

**2. Results**

2.1 The chemical fingerprints of *Rheum officinale* Baill. were demonstrated in the **Supplementary Figures 1 and 2**. And the specific ingredients of it are indicated in **Supplementary Tables 1 and 2**.


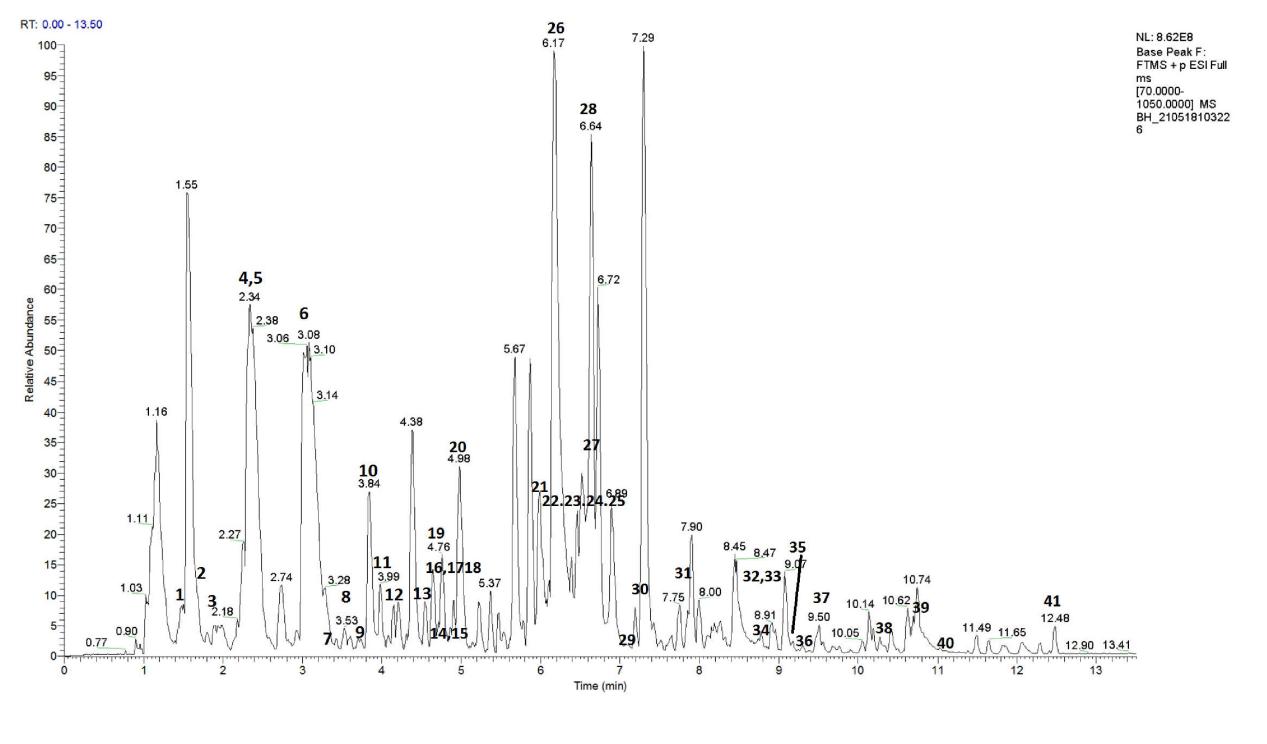


**Supplementary Figure 1** The UHPLC-Q-Orbitrap HRMS chemical fingerprint of *Rheum officinale* Baill. (pos-pbc).


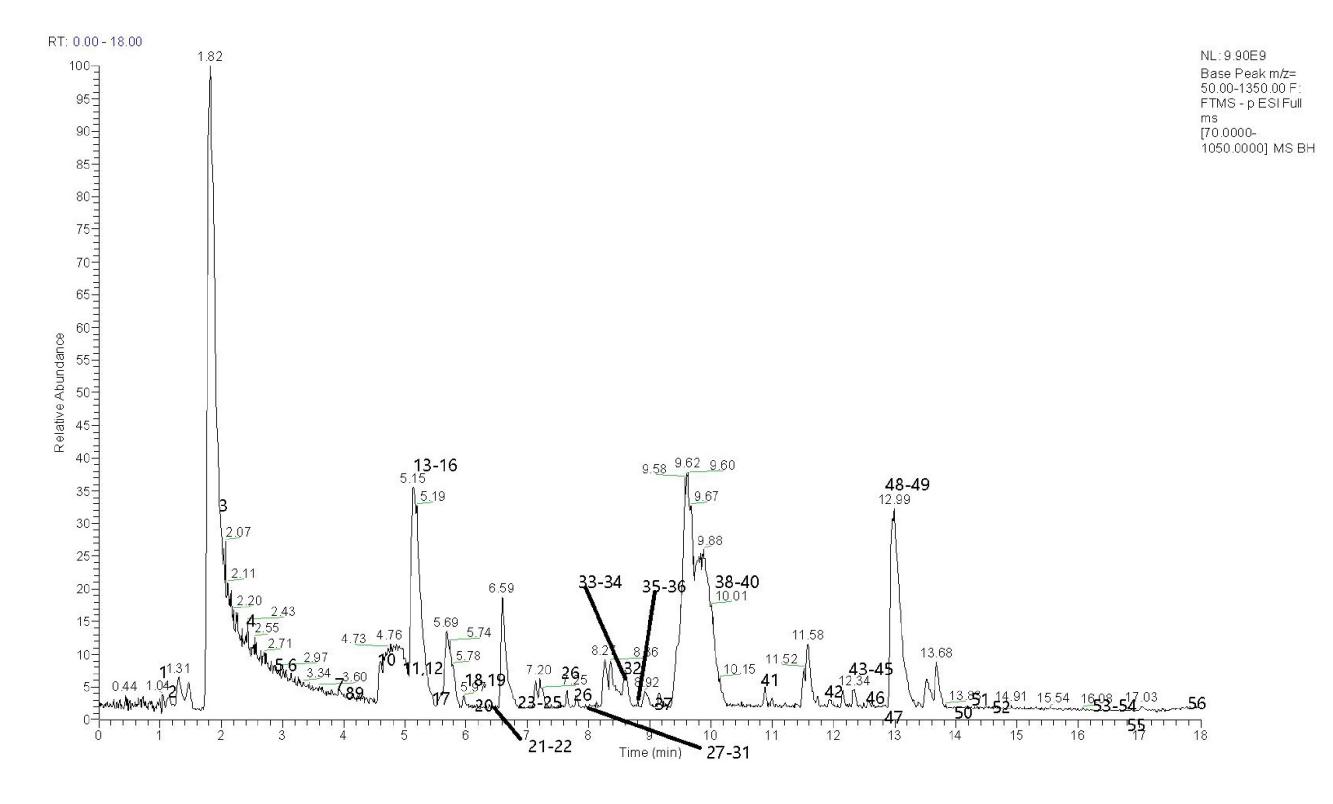


**Supplementary Figure 2** The UHPLC-Q-Orbitrap HRMS chemical fingerprint of *Rheum officinale* Baill. (neg-pbc).

**Supplementary Table 1** UHPLC-Q-Orbitrap HRMS chemical fingerprint (pos-pbc) ingredients.

| **Index** | **Compound** | **Corrected tR(s)** | **CAS No.** | **Molecular Formula** |
| --- | --- | --- | --- | --- |
| 1 | Epigallo catechin | 85.23478 | 970-74-1 | C_15_H_14_O_7_ |
| 2 | Catechin 7-glucoside | 100.46453 | 65597-47-9 | C_21_H_24_O_11_ |
| 3 | Epicatechin 3-glucoside | 115.20292 | 103303-00-0 | C_21_H_24_O_11_ |
| 4 | Fisetinidol-4β-ol | 138.61743 | 967-30-6 | C_15_H_14_O_6_ |
| 5 | epicatechin 8-C-β-D-glucopyranoside | 138.61743 | / | / |
| 6 | (+)-Epicatechin | 184.16746 | 35323-91-2 | C_15_H_14_O_6_ |
| 7 | Puerarin | 208.34321 | 3681-99-0 | C_21_H_20_O_9_ |
| 8 | Swertiamarin | 211.87028 | 17388-39-5 | C_16_H_22_O_10_ |
| 9 | 5-Hydroxy-7-methoxy-6,8-di-C-methylflavanone | 224.07896 | / | C_18_H_18_O_4_ |
| 10 | Vanillic acid | 228.43465 | 121-34-6 | C_8_H_8_O_4_ |
| 11 | α-Hydrojuglone 4-O-b-D-glucoside | 240.55046 | 39015-63-9 | C_16_H_18_O_8_ |
| 12 | 1-Caffeoylquinic Acid | 247.75984 | / | C_16_H_18_O_9_ |
| 13 | Protoanemoni | 269.21476 | 108-28-1 | C_5_H_4_O_2_ |
| 14 | Chrysosplenetin | 274.26805 | 603-56-5 | C_19_H_18_O_8_ |
| 15 | 2-Hydroxy-8-methylchromene-2-carboxylate | 274.98229 | 156605-21-9 | C_11_H_10_O_4_ |
| 16 | Gambiriin C | 277.19525 | 76236-89-0 | C_30_H_26_O_11_ |
| 17 | Flavanone 7-O-β-D-glucoside | 278.63336 | / | C_21_H_22_O_8_ |
| 18 | Chrysophanic acid 9-anthrone | 278.63336 | 491-58-7 | C_15_H_12_O_3_ |
| 19 | 3,3',4',5,7-Pentahydroxyflavan(4->8)-3,4',5,7-tetrahydroxyflavan | 285.75626 | 80685-13-8 | C_30_H_26_O_11_ |
| 20 | Pyrogallinp | 291.48991 | 18483-80-2 | C_11_H_8_O_4_ |
| 21 | Geniposidic acid | 299.34854 | 27741-01-1 | C_16_H_22_O_10_ |
| 22 | Esculetin | 363.69314 | 305-01-1 | C_9_H_6_O_4_ |
| 23 | Peonidin 3-O-glucoside | 365.06525 | 68795-37-9 | C_22_H_23_O_11_ |
| 24 | Isolindleyin | 365.06525 | 87075-18-1 | C_23_H_26_O_11_ |
| 25 | Gallate | 365.06525 | 149-91-7 | C_7_H_6_O_5_ |
| 26 | Apigenin 7-(2''-glucosyllactate) | 369.97606 | / | C_24_H_24_O_12_ |
| 27 | Sennoside A & B | 393.93716 | / | / |
| 28 | Chrysophanol | 411.5599 | 481-74-3 | C_15_H_10_O_4_ |
| 29 | Physcion 8-glucoside | 424.31457 | 26296-54-8 | C_22_H_22_O_10_ |
| 30 | 3-O-Acetylpinobanksin | 427.75778 | 52117-69-8 | / |
| 31 | Rheinoside A | 469.11824 | / | / |
| 32 | Swertiajaponin | 508.89961 | 6980-25-2 | C_22_H_22_O_11_ |
| 33 | Emodin 8-glucoside | 509.62099 | 38840-23-2 | C_21_H_20_O_10_ |
| 34 | Citreorosein | 518.86471 | 481-73-2 | C_15_H_10_O_6_ |
| 35 | Rhein diglucoside | 548.06175 | / | / |
| 36 | Aloe-emodin | 550.9477 | 481-72-1 | C_15_H_10_O_5_ |
| 37 | Chrysin | 572.42426 | 480-40-0 | C_15_H_10_O_4_ |
| 38 | 3',4',7-Trihydroxyisoflavone | 615.29706 | 485-63-2 | C_15_H_10_O_5_ |
| 39 | Rhein | 641.00052 | 478-43-3 | C15H8O6 |
| 40 | Physcion | 674.43978 | 521-61-9 | C_16_H_12_O_5_ |
| 41 | Emodin | 748.68138 | 518-82-1 | C_15_H_10_O_5_ |

* / indicating the related information is not acquired.

**Supplementary Table 2** UHPLC-Q-Orbitrap HRMS chemical fingerprint (neg-pbc) ingredients.

| **Index** | **Compound** | **Corrected tR(s)** | **CAS No.** | **Molecular Formula** |
| --- | --- | --- | --- | --- |
| 1 | Vanillic acid | 65.81024 | 121-34-6 | C_8_H_8_O_4_ |
| 2 | Phlorin | 71.14453 | 28217-60-9 | C_12_H_16_O_8_ |
| 3 | Shanzhiside methyl ester | 113.86199 | / | C_17_H_26_O_11_ |
| 4 | 2,6-Digalloylglucose | 145.36073 | 94356-20-4 | C_20_H_20_O_14_ |
| 5 | Ellagic acid | 174.63916 | 476-66-4 | C_14_H_6_O_8_ |
| 6 | Fisetinidol | 180.07895 | 490-49-3 | C_15_H_14_O_5_ |
| 7 | (-)-Epicatechin 7-O-glucuronide | 224.87462 | / | C_21_H_22_O_12_ |
| 8 | Semilepidinoside B | 243.21383 | / | C_17_H_22_N_2_O_7_ |
| 9 | Procyanidin B4 | 257.62336 | 4852-22-6 | C_30_H_26_O_12_ |
| 10 | 4-Vinylguaiacol | 276.14833 | 7786-61-0 | C_9_H_10_O_2_ |
| 11 | Aloe emodin w-acetate | 291.79596 | 65615-58-9 | C_17_H_12_O_6_ |
| 12 | Gallic acid 4-O-(6-galloylglucoside) | 295.17715 | 87087-62-5 | C_20_H_20_O_14_ |
| 13 | Sennoside A | 306.15323 | 81-27-6 | C_42_H_38_O_20_ |
| 14 | n-Propyl gallate | 307.51645 | 121-79-9 | C_10_H_12_O_5_ |
| 15 | Catechin | 308.85392 | 154-23-4 | C_15_H_14_O_6_ |
| 16 | Catechin 5-glucoside | 312.23337 | 88126-53-8 | C_21_H_24_O_11_ |
| 17 | Salidroside | 328.49849 | 10338-51-9 | C_14_H_20_O_7_ |
| 18 | Glucorhein | 353.011 | 34298-86-7 | C_21_H_18_O_11_ |
| 19 | 2,4-Dihydroxyacetophenone 5-sulfate | 358.46027 | / | C_8_H_8_O_6_S |
| 20 | Cynaroside | 368.87373 | 5373/11/5 | C21H20O11 |
| 21 | Catechin 3'-glucoside | 380.45479 | 105330-51-6 | C_21_H_24_O_11_ |
| 22 | Catechin 7-O-β-D-xyloside | 389.97554 | 65597-47-9 | C_20_H_22_O_10_ |
| 23 | Pelargonin | 415.88987 | 17334-58-6 | C_27_H_31_O_15_ |
| 24 | Purpurogallin | 424.01951 | 569-77-7 | C_11_H_8_O_5_ |
| 25 | Eriodictyol | 428.66029 | 4049-38-1 | C_15_H_12_O_6_ |
| 26 | Phloridzin | 449.85133 | 7061-54-3 | C_21_H_24_O_10_ |
| 27 | Homovanillic acid sulfate | 459.4197 | 38339-06-9 | C_9_H_10_O_7_S |
| 28 | Isolindleyin | 468.31879 | 87075-18-1 | C_23_H_26_O_11_ |
| 29 | 3-Hydroxyphloretin | 471.71074 | / | C_15_H_14_O_6_ |
| 30 | Vitexin | 483.21495 | 3681-93-4 | C_21_H_20_O_10_ |
| 31 | Cinchonain Id 7-glucoside | 484.54379 | / | C_30_H_30_O_14_ |
| 32 | (-)-Epiafzelechin | 507.3658 | 24808-04-6 | C_15_H_14_O_5_ |
| 33 | Genistein 8-C-glucoside | 514.78331 | 66026-80-0 | C_21_H_20_O_10_ |
| 34 | Citreorosein | 518.1865 | 481-73-2 | C_15_H_10_O_6_ |
| 35 | Isopimpinellin | 528.97747 | 482-27-9 | C_13_H_10_O_5_ |
| 36 | 2-(3,4-dihydroxyphenyl)-3,5,6,7-tetrahydroxy-4H-chromen-4-one | 530.40719 | / | C_15_H_10_O_8_ |
| 37 | Eugenin | 542.56063 | 480-34-2 | C_11_H_10_O_4_ |
| 38 | Fisetin | 596.37853 | 528-48-3 | C_15_H_10_O_6_ |
| 39 | Chrysophanol 8-gentiobioside | 597.06266 | 54944-38-6 | C_27_H_30_O_14_ |
| 40 | Xanthopurpurin | 599.81585 | 518-83-2 | C_14_H_8_O_4_ |
| 41 | Apigenin O-glucoside malonylated | 637.13262 | / | C_24_H_22_O_13_ |
| 42 | Pelargonidin 3-O-glucoside | 716.94438 | 18466-51-8 | C_21_H_21_O_10_ |
| 43 | Apigenin 7-sulfate | 740.54316 | 56857-56-8 | C_15_H_10_O_8_S |
| 44 | Chrysophanol 8-O-β-D-glucoside | 741.90264 | 13241-28-6 | C_21_H_20_O_9_ |
| 45 | Chrysin | 742.61136 | 480-40-0 | C_15_H_10_O_4_ |
| 46 | 3',4,4'-Trihydroxypulvinone | 755.1264 | 51282-12-3 | C_17_H_12_O_6_ |
| 47 | Curcumin II | 770.37978 | 91884-87-6 | C_22_H_22_O_5_ |
| 48 | Dihydrocoriandrin | 774.55938 | 116383-99-4 | C_13_H_12_O_4_ |
| 49 | Apigenin | 779.31816 | 520-36-5 | C_15_H_10_O_5_ |
| 50 | Genistein | 837.3849 | 446-72-0 | C_15_H_10_O_5_ |
| 51 | Aloe-emodin | 848.40108 | 481-72-1 | C_15_H_10_O_5_ |
| 52 | 3-Methylgalangin | 871.81806 | 6665-74-3 | C_16_H_12_O_5_ |
| 53 | (-)-Epigallocatechin 3'-glucuronide | 981.62088 | / | C_21_H_22_O_13_ |
| 54 | (-)-Epigallocatechin 3'-glucuronide | 981.62088 | / | C_21_H_22_O_13_ |
| 55 | Malvidin 3-O-glucoside | 1015.4238 | 18470-06-9 | C_23_H_25_O_12_ |
| 56 | Glycitein | 1075.85376 | 40957-83-3 | C_16_H_12_O_5_ |

* / indicating the related information is not acquired.

2.2 The XFP chemical fingerprints were showed in the **Supplementary Figures 3 and 4**. And the specific ingredients of it are indicated in **Supplementary Tables 3 and 4**.


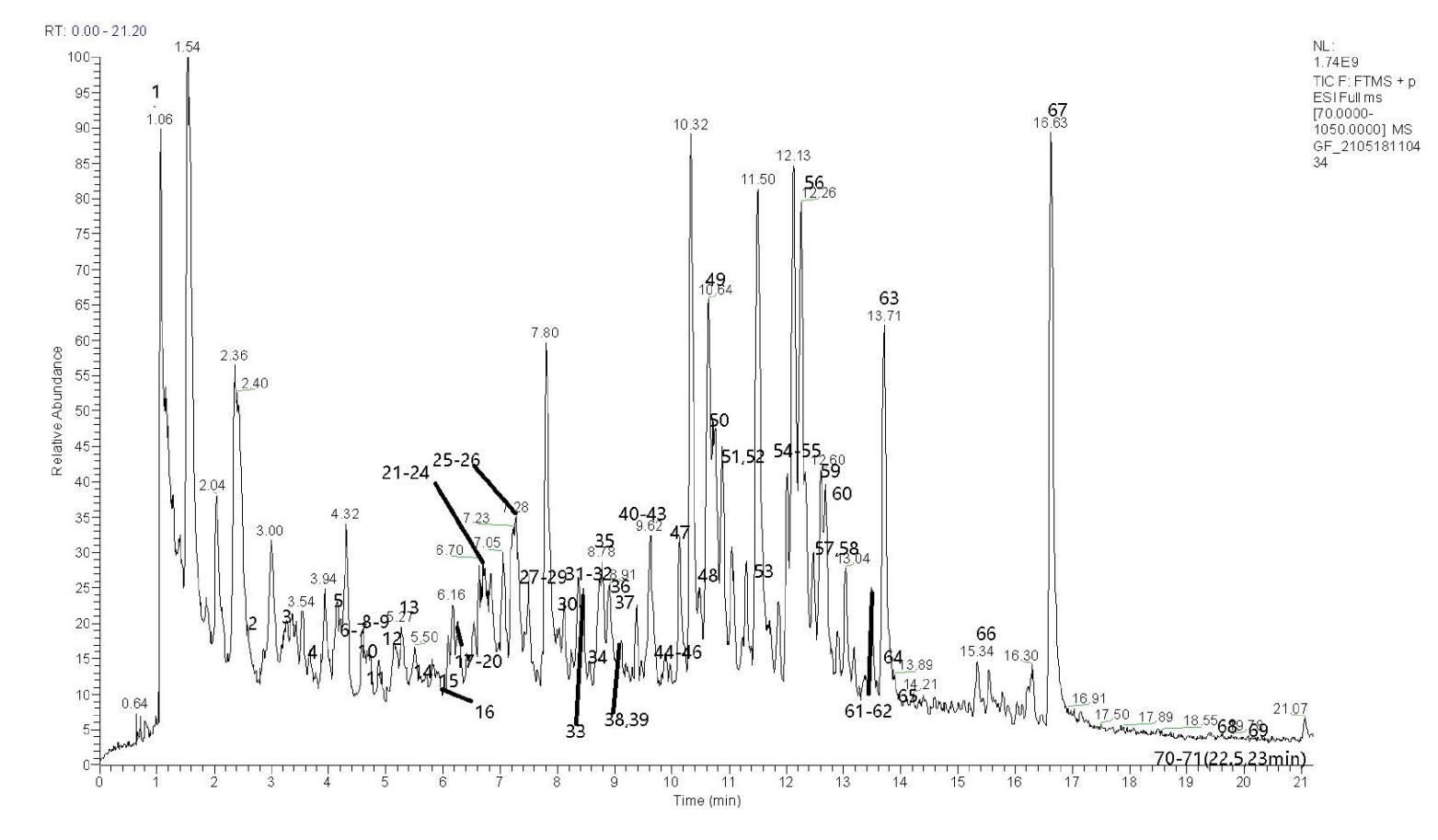


**Supplementary Figure 3** The UHPLC-Q-Orbitrap HRMS chemical fingerprint of XFP (pos-pbc).

**
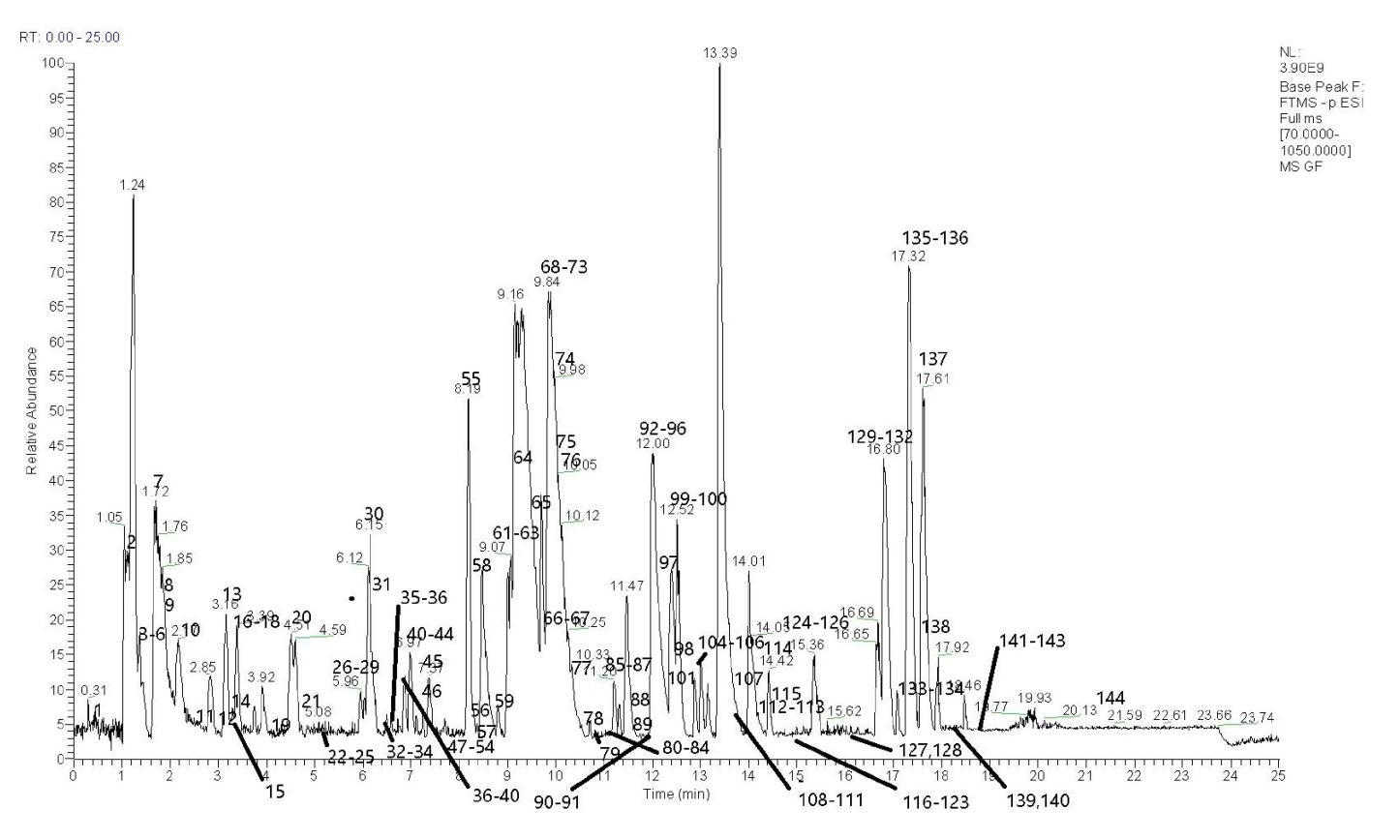
**

**Supplementary Figure 4** The UHPLC-Q-Orbitrap HRMS chemical fingerprint of XFP (neg-pbc).

**Supplementary Table 3** UHPLC-Q-Orbitrap HRMS chemical fingerprint (pos-pbc) compoments.

| **Index** | **Ingredient** | **Corrected tR(s)** | **CAS No.** | **Molecular Formula** |
| --- | --- | --- | --- | --- |
| 1 | Hordenine | 1.10 | 539-15-1 | C_10_H_15_NO |
| 2 | Cichoriin | 2.56 | 531-58-8 | C_15_H_16_O_9_ |
| 3 | trans-Ferulic Acid | 3.20 | 537-98-4 | / |
| 4 | Glucovanillin | 3.72 | 494-08-6 | C_14_H_18_O_8_ |
| 5 | Syringin | 4.13 | 118-34-3 | C_17_H_24_O_9_ |
| 6 | Multinoside A | 4.24 | / | C_27_H_30_O_16_ |
| 7 | 1. [(2S,3R,4S,5S,6R)-3,4, 2. 5-trihydroxy-6-(hydroxymethyl) 3. oxan-2-yl]oxychromen-2-one | 4.28 | / | C_15_H_16_O_8_ |
| 8 | Esculetin | 4.57 | 305-01-1 | C_9_H_6_O_4_ |
| 9 | Daphnin | 4.58 | 486-55-5 | C_15_H_16_O_9_ |
| 10 | Kaempferol-3-O-glucosyl(1-2)  rhamnoside | 4.75 | / | C_27_H_30_O_15_ |
| 11 | Staphylin | 4.88 | / | C_16_H_18_O_9_ |
| 12 | Isorhamnetin-3-O-Neohespeidoside | 5.14 | / | C_28_H_32_O_16_ |
| 13 | Cirsimarin | 5.33 | 13020-19-4 | C_23_H_24_O_11_ |
| 14 | Isovitexin 2''-O-Arabinoside | 5.66 | / | C_26_H_28_O_14_ |
| 15 | Laricitrin 3-galactoside | 5.89 | / | C_22_H_22_O_13_ |
| 16 | Rutin | 6.00 | 153-18-4 | C_27_H_30_O_16_ |
| 17 | Isorhamnetin-3-O-rutinoside | 6.39 | 604-80-8 | C_28_H_32_O_16_ |
| 18 | Mahaleboside | 6.48 | / | C_15_H_16_O_8_ |
| 19 | Apigenin 7-O-neohesperidoside | 6.51 | 17306-46-6 | C_27_H_30_O_14_ |
| 20 | Isorhamnetin 3-glucoside | 6.58 | / | C_22_H_22_O_12_ |
| 21 | Diosmin | 6.69 | 520-27-4 | C_28_H_32_O_15_ |
| 22 | Naringin | 6.73 | / | C_27_H_32_O_14_ |
| 23 | [Narirutin](https://pubchem.ncbi.nlm.nih.gov/compound/442431) | 6.73 | / | C_27_H_32_O_14_ |
| 24 | Keioside | 6.81 | 107740-46-5 | C_28_H_32_O_16_ |
| 25 | Hesperidin | 7.25 | 520-26-3 | C_28_H_34_O_15_ |
| 26 | Syringetin-3-O-glucoside | 7.28 | 40039-49-4 | C_23_H_24_O_13_ |
| 27 | 6''-O-(3-Hydroxy-3-methylglutaroyl)  astragalin | 7.54 | 157407-84-6 | C_27_H_28_O_15_ |
| 28 | Bis-noryangonin | 7.65 | 13709-27-8 | C_13_H_10_O_4_ |
| 29 | Eriodictyol 7,3'-dimethyl ether | 7.65 | 54352-60-2 | / |
| 30 | Resveratrol | 7.99 | 501-36-0 | C_14_H_12_O_3_ |
| 31 | Centaurein | 8.20 | / | C_24_H_26_O_13_ |
| 32 | Biochanin A | 8.29 | 491-80-5 | C_16_H_12_O_5_ |
| 33 | Isoswertisin 2''-rhamnoside | 8.42 | 64821-00-7 | C_28_H_32_O_14_ |
| 34 | Malvidin 3-(6''-acetylglucoside) | 8.62 | / | C_25_H_26_O_13_ |
| 35 | 5,7,3'-Trihydroxy-3,6,4',5'-  tetramethoxyflavone | 8.74 | / | C_19_H_18_O_9_ |
| 36 | Naringerin | 8.91 | 67604-48-2 | C_15_H_12_O_5_ |
| 37 | Diosmetin | 8.98 | 520-34-3 | C_16_H_12_O_6_ |
| 38 | Hesperetin | 9.38 | 520-33-2 | C_16_H_14_O_6_ |
| 39 | Myricetin 3,7,3',4'-tetramethyl ether | 9.48 | / | C_19_H_18_O_8_ |
| 40 | Fraxetin | 9.54 | 574-84-5 | C_10_H_8_O_5_ |
| 41 | (-)-BEBEERINE | 9.55 | / | / |
| 42 | (S)-Auraptenol | 9.62 | 1221-43-8 | C_15_H_16_O_4_ |
| 43 | Angelicin | 9.63 | 523-50-2 | C_11_H_6_O_3_ |
| 44 | Alantolactone | 9.87 | 546-43-0 | C_15_H_20_O_2_ |
| 45 | Isopimpinellin | 9.94 | 482-27-9 | C_13_H_10_O_5_ |
| 46 | Liquiritigenin 7-apiofuranoside-4'-glucoside | 9.99 | / | C_26_H_30_O_13_ |
| 47 | Tangeretin | 10.13 | 481-53-8 | C_20_H_20_O_7_ |
| 48 | Sinensetin | 10.44 | / | / |
| 49 | Isobergaptol | 10.61 | / | C_11_H_6_O_4_ |
| 50 | (-)-Usnic acid | 10.65 | 6159-66-6 | C_18_H_16_O_7_ |
| 51 | Umbelliferone | 10.94 | 93-35-6 | C_9_H_6_O_3_ |
| 52 | MULTIFLORASIDE | 11.02 | / | C_21_H_22_O_10_ |
| 53 | 4',5,6,7-Tetramethoxyflavone | 11.45 | 1168-42-9 | C_19_H_18_O_6_ |
| 54 | Casticin | 11.89 | 479-91-4 | C_19_H_18_O_8_ |
| 55 | Nobiletin | 11.91 | 478-01-3 | C_21_H_22_O_8_ |
| 56 | Justicidin A | 12.28 | 25001-57-4 | C_22_H_18_O_7_ |
| 57 | Naringenin chalcone | 12.40 | 5071-40-9 | C_15_H_12_O_5_ |
| 58 | Homonataloin B | 12.42 | / | C_22_H_24_O_9_ |
| 59 | GLYCOPERINE | 12.60 | / | C_19_H_21_NO_8_ |
| 60 | α-Amyrone | 12.85 | 638-96-0 | C_30_H_48_O |
| 61 | Betulin | 13.59 | 473-98-3 | C_30_H_50_O_2_ |
| 62 | Lupenone | 13.59 | 1617-70-5 | C_30_H_48_O |
| 63 | Bergaptol | 13.71 | 486-60-2 | C_11_H_6_O_4_ |
| 64 | β-Amyrone | 13.80 | / | C_30_H_48_O |
| 65 | Soyaspongenol C | 14.07 | / | / |
| 66 | Coroglaucigenin-3-o-α-L-rhamnopyranoside | 15.43 | / | C_29_H_44_O_9_ |
| 67 | 7-Prenylumbelliferone | 16.63 | / | / |
| 68 | Auraptene | 19.41 | 495-02-3 | C_19_H_22_O_3_ |
| 69 | β-Sitosterol | 20.34 | 83-46-5 | C_29_H_50_O |
| 70 | Stigmasterol | 22.46 | 83-48-7 | C_29_H_48_O |
| 71 | Campesterol | 22.86 | 474-62-4 | C_28_H_48_O |

* / indicating the related information is not acquired.

**Supplementary Table 4** UHPLC-Q-Orbitrap HRMS chemical fingerprint (neg-pbc) compoments.

| **Index** | **Ingredient** | **Corrected tR(s)** | **CAS No.** | **Molecular Formula** |
| --- | --- | --- | --- | --- |
| 1 | Sinapoyl aldehyde | 0.44 | 4206-58-0 | C_11_H_12_O_4_ |
| 2 | Catechin | 1.09 | 154-23-4 | C_15_H_14_O_6_ |
| 3 | 2-(4-Hydroxyphenl)-Ethonol | 1.43 | 501-94-0 | C_8_H_10_O_2_ |
| 4 | 2-Hydroxy cinnamic acid | 1.43 | 614-60-8 | C_9_H_8_O_3_ |
| 5 | Phloroglucinol carboxylic acid | 1.52 | 83-30-7 | C_7_H_6_O_5_ |
| 6 | 2',4'-Dihydroxy-6'-methoxy-3',5'-dimethylchalcone | 1.60 | 65349-31-7 | C_18_H_18_O_4_ |
| 7 | Nicotinamide | 1.70 | 98-92-0 | C_6_H_6_N_2_O |
| 8 | Phlorin | 1.98 | 28217-60-9 | C_12_H_16_O_8_ |
| 9 | Synephrine | 2.01 | 1994/7/5 | C_9_H_13_NO_2_ |
| 10 | Scytalone | 2.22 | 49598-85-8 | C_10_H_10_O_4_ |
| 11 | Citrusin F | 2.72 | 134860-04-1 | C_22_H_32_O_14_ |
| 12 | 5-O-Caffeoylshikimic acid | 2.96 | 73263-62-4 | C_16_H_16_O_8_ |
| 13 | Pratensein 3'-glucoside | 3.16 | / | C_22_H_22_O_11_ |
| 14 | Licoagroside B | 3.24 | / | C_18_H_24_O_12_ |
| 15 | Lysyl-Isoleucine | 3.26 | / | C_12_H_25_N_3_O_3_ |
| 16 | Neocarthamin | 3.37 | / | C_21_H_22_O_11_ |
| 17 | 5,7-Dihydroxychromone | 3.39 | 31721-94-5 | C_9_H_6_O_4_ |
| 18 | Iprobenfos | 3.45 | 26087-47-8 | C_13_H_21_O_3_PS |
| 19 | Semilepidinoside B | 4.16 | / | C_17_H_22_N_2_O_7_ |
| 20 | Eleutheroside B1 | 4.50 | 16845-16-2 | C_17_H_20_O_10_ |
| 21 | Phloridzin | 4.68 | 7061-54-3 | C_21_H_24_O_10_ |
| 22 | Isomangiferin | 5.21 | 24699-16-9 | C_19_H_18_O_11_ |
| 23 | 2'-Hydroxygenistein 8-C-glucoside; 2'-Hydroxygenistein-8-C-glucoside | 5.44 | / | C_21_H_20_O_11_ |
| 24 | Salidroside | 5.45 | 10338-51-9 | C_14_H_20_O_7_ |
| 25 | 5,7,8-Trihydroxyflavanone 7-glucoside | 5.51 | / | C_21_H_22_O_10_ |
| 26 | Kaempferol 3-O-β-rutinoside | 6.00 | 17650-84-9 | C_27_H3_0_O15 |
| 27 | Adicardin | 6.01 | / | C_20_H_24_O_12_ |
| 28 | 5-hydroxy-2-[1-hydroxy-4-[(2R,3R,4S,5S,6R)-3,4,5-trihydroxy-6-(hydroxymethyl)oxan-2-yl]oxycyclohexa-2,5-dien-1-yl]-7-methoxychromen-4-one | 6.04 | / | C_22_H_24_O_11_ |
| 29 | Ferreirin | 6.04 | 32898-79-6 | C_16_H_14_O_6_ |
| 30 | Genistein 8-C-glucoside | 6.17 | 66026-80-0 | C_21_H_20_O_10_ |
| 31 | Vicenin 2 | 6.20 | / | C_27_H_30_O_15_ |
| 32 | Miscanthoside | 6.34 | 38965-51-4 | C_21_H_22_O_11_ |
| 33 | Laricitrin 3-glucoside | 6.36 | / | C_22_H_22_O_13_ |
| 34 | (E)-Oxyresveratrol 3'-O-b-D-glucoside | 6.37 | 144525-40-6 | C_20_H_22_O_9_ |
| 35 | Sinapyl alcohol | 6.52 | 537-33-7 | C_11_H_14_O_4_ |
| 36 | naringenin-7-O-glucoside | 6.55 | / | C_21_H_22_O_10_ |
| 37 | 3-[(2S,3R,4S,5R,6S)-3,4-dihydroxy-6-methyl-5-[(2S,3R,4S,5S,6R)-3,4,5-trihydroxy-6-(hydroxymethyl)oxan-2-yl]oxyoxan-2-yl]oxy-2-(3,4-dihydroxyphenyl)-5-hydroxy-7-[3,4,5-trihydroxy-6-(hydroxymethyl)oxan-2-yl]oxychromen-4-one | 6.82 | / | C_33_H_40_O_21_ |
| 38 | Daphnetin | 6.86 | / | C_9_H_6_O_4_ |
| 39 | Esculetin | 6.87 | 305-01-1 | C_9_H_6_O_4_ |
| 40 | Scolymoside | 6.87 | 25694-72-8 | C_27_H_30_O_15_ |
| 41 | Vitexin | 6.90 | 3681-93-4 | C_21_H_20_O_10_ |
| 42 | 1-O-Sinapoylglucose | 6.92 | / | C_17_H_22_O_10_ |
| 43 | 4-METHYLDAPHNETIN | 7.05 | 2107-77-9 | C_10_H_8_O_4_ |
| 44 | Eriodictyol | 7.08 | 4049-38-1 | C_15_H_12_O_6_ |
| 45 | Zingerone glucoside | 7.35 | 64703-96-4 | C_17_H_24_O_8_ |
| 46 | Trilobatin | 7.43 | 4192-90-9 | C_21_H_24_O_10_ |
| 47 | Hesperetin 5-O-glucoside | 7.70 | 69651-80-5 | C_22_H_24_O_11_ |
| 48 | 1-(2,6-Dihydroxy-4-methoxyphenyl)ethanone | 7.74 | 7507-89-3 | C_9_H_10_O_4_ |
| 49 | Petunidin 3-O-glucoside | 7.81 | 6988-81-4 | C_22_H_23_O_12_ |
| 50 | 4',8-Dimethylgossypetin 3-glucoside | 7.82 | 90456-57-8 | C_23_H_24_O_13_ |
| 51 | 6-Epi-7-isocucurbic acid glucoside | 7.92 | 176486-13-8 | C_18_H_30_O_8_ |
| 52 | Limocitrin 3-glucoside | 7.99 | 38836-51-0 | C_23_H_24_O_13_ |
| 53 | Centaurein | 8.04 | 35595-03-0 | C_24_H_26_O_13_ |
| 54 | Celereoin | 8.04 | 74560-02-4 | C_14_H_14_O_5_ |
| 55 | Naringerin | 8.19 | 67604-48-2 | C_15_H_12_O_5_ |
| 56 | Rutin | 8.35 | 153-18-4 | C_27_H_30_O_16_ |
| 57 | 3',5,7-Trihydroxy-3'-methoxyflavanone, Homoeriodictyol | 8.49 | / | C_16_H_14_O_6_ |
| 58 | 2'-Hydroxydihydrodaidzein | 8.51 | / | C_15_H_12_O_5_ |
| 59 | Loquatoside | 8.75 | 74046-15-4 | C_20_H_22_O_11_ |
| 60 | Icariside B8 | 8.83 | 126176-78-1 | C_19_H_32_O_8_ |
| 61 | Distichonic acid B | 9.00 | 84495-21-6 | C_10_H_18_N_2_O_8_ |
| 62 | Haematoxylin | 9.01 | 517-28-2 | C_16_H_14_O_6_ |
| 63 | MULTIFLORASIDE | 9.10 | / | C_21_H_22_O_10_ |
| 64 | D-Tagatose | 9.26 | 87-81-0 | C_6_H_12_O_6_ |
| 65 | 7-[4,5-dihydroxy-6-(hydroxymethyl)-3-[(2S,3R,4R,5R,6S)-3,4,5-trihydroxy-6-methyloxan-2-yl]oxyoxan-2-yl]oxy-5-hydroxy-2-(4-hydroxyphenyl)-2,3-dihydrochromen-4-one | 9.41 | / | C_27_H_32_O_14_ |
| 66 | Arbutin | 9.53 | 497-76-7 | C_12_H_16_O_7_ |
| 67 | 3,6-Dimethoxyapigenin | 9.55 | / | / |
| 68 | Hesperetin 7-(2,6-dirhamnosylglucoside) | 9.66 | 97218-30-9 | C_34_H_44_O_19_ |
| 69 | Genistein | 9.69 | 446-72-0 | C_15_H_10_O_5_ |
| 70 | Epihesperidin | 9.70 | 369593-42-0 | C_28_H_34_O_15_ |
| 71 | Nomilinic acid | 9.74 | 35930-20-2 | C_28_H_36_O_10_ |
| 72 | Hesperetin 7-O-glucoside | 9.80 | 2500-68-7 | C_22_H_24_O_11_ |
| 73 | Dihydromethysticin | 9.81 | 19902-91-1 | C_15_H_16_O_5_ |
| 74 | Keioside | 9.93 | 107740-46-5 | C_28_H_32_O_16_ |
| 75 | (1β,4α,5α,6β,8α,10b)-1,10:4,5-Diepoxy-6-hydroxy-7(11)-germacren-12,8-olide | 10.02 | 366494-94-2 | C_15_H_20_O_5_ |
| 76 | Naringenin | 10.04 | 480-41-1 | C_15_H_12_O_5_ |
| 77 | Lactucin | 10.30 | 1891-29-8 | C_15_H_16_O_5_ |
| 78 | 1-(2-Methoxy-3,4-methylenedioxyphenyl)-1-propanone | 10.62 | / | C_11_H_12_O_4_ |
| 79 | Dihydrogenistein | 10.79 | 21554-71-2 | C_15_H_12_O_5_ |
| 80 | Hydroxymyricanone | 11.00 | / | C_21_H_24_O_6_ |
| 81 | Garbanzol | 11.03 | 1226-22-8 | C_15_H_12_O_5_ |
| 82 | Terretonin | 11.03 | 71911-90-5 | C_26_H_32_O_9_ |
| 83 | Naringin | 11.07 | / | C_27_H_32_O_14_ |
| 84 | 11-nitro-1-undecene | 11.10 | / | C_11_H_21_NO_2_ |
| 85 | Torachrysone | 11.22 | 22649-04-3 | C_14_H_14_O_4_ |
| 86 | tiliroside | 11.28 | / | C_30_H_26_O_13_ |
| 87 | β-D-Glucosyl crocetin | 11.32 | 58050-17-2 | C_26_H_34_O_9_ |
| 88 | Didymin | 11.49 | / | C_28_H_34_O_14_ |
| 89 | Diosmetin | 11.58 | 520-34-3 | C_16_H_12_O_6_ |
| 90 | Nodakenetin | 11.74 | 13849-08-6 | C_14_H_14_O_4_ |
| 91 | (R)-Marmin | 11.78 | 14957-38-1 | C_19_H_24_O_5_ |
| 92 | Corchoroside B | 11.86 | 35536-76-6 | C_29_H_42_O_8_ |
| 93 | Arctigenin | 11.87 | 7770-78-7 | C_21_H_24_O_6_ |
| 94 | Dihydromyoporone | 11.95 | 72145-16-5 | C_15_H_24_O_3_ |
| 95 | Imperatorin | 12.01 | 482-44-0 | C_16_H_14_O_4_ |
| 96 | Torachrysone 8-(2-apiosylglucoside) | 12.02 | 245724-11-2 | C_25_H_32_O_13_ |
| 97 | Secoisolariciresinol | 12.20 | 29388-59-8 | C_20_H_26_O_6_ |
| 98 | Scutellarein 4'-methyl ether | 12.25 | / | C_16_H_12_O_6_ |
| 99 | Austalide E | 12.40 | 81543-05-7 | C_28_H_36_O_10_ |
| 100 | 2'-O-Methylisoliquiritigenin | 12.54 | 51828-10-5 | C_16_H_14_O_4_ |
| 101 | Narirutin | 12.74 | 14259-46-2 | C_27_H_32_O_14_ |
| 102 | Nomilin | 13.02 | 1063-77-0 | C_28_H_34_O_9_ |
| 103 | 5,7-dihydroxy-2-(3-hydroxy-4,5-dimethoxyphenyl)-3,6-dimethoxy-4H-chromen-4-one | 13.03 | / | / |
| 104 | Duartin, Dimethyl Ether | 13.11 | / | C_20_H_24_O_6_ |
| 105 | Cyperine | 13.23 | 33716-82-4 | C_15_H_16_O_4_ |
| 106 | (-)-Usnic acid | 13.27 | 6159-66-6 | C_18_H_16_O_7_ |
| 107 | Poncirin | 13.56 | 14941-08-3 | C_28_H_34_O_14_ |
| 108 | Isofraxidin | 13.63 | 486-21-5 | C_11_H_10_O_5_ |
| 109 | Perlolyrine | 13.69 | 29700-20-7 | C_16_H_12_N_2_O_2_ |
| 110 | Cristacarpin | 13.75 | 74515-47-2 | C_21_H_22_O_5_ |
| 111 | Eupatilin | 13.87 | / | C_18_H_16_O_7_ |
| 112 | Ginsenoside Rg1 | 14.19 | 22427-39-0 | C_42_H_72_O_14_ |
| 113 | Bupivacaine | 14.20 | 2180-92-9 38396-39-3 | C_18_H_28_N_2_O |
| 114 | Xanthotoxol | 14.43 | 2009-24-7 | C_11_H_6_O_4_ |
| 115 | Scopoletin | 14.49 | 92-61-5 | C_10_H_8_O_4_ |
| 116 | Squamostanal A | 14.63 | 156764-90-8 | C_18_H_30_O_3_ |
| 117 | Ginsenoside Rg3 | 14.87 | 14197-60-5 | C_42_H_72_O_13_ |
| 118 | 2',4,4'-Trihydroxy-6'-methoxy-3',5'-diprenylchalcone | 14.87 | 189299-04-5 | C_26_H_30_O_5_ |
| 119 | 7-hydroxy-coumarin | 14.99 | 93-35-6 | / |
| 120 | Curdione | 15.02 | 13657-68-6 | C_15_H_24_O_2_ |
| 121 | 16,16-dimethyl-6-keto Prostaglandin E1 | 15.06 | 75874-32-7 | C_22_H_36_O_6_ |
| 122 | Scandoside methyl ester | 15.15 | / | C_17_H_24_O_11_ |
| 123 | Lubimin | 15.17 | 64024-09-5 | C_15_H_24_O_2_ |
| 124 | Arteanoflavone | 15.35 | / | / |
| 125 | Hernandulcin | 15.35 | 95602-94-1 | C_15_H_24_O_2_ |
| 126 | Cynaroside A | 15.46 | 117804-06-5 | C_21_H_32_O_10_ |
| 127 | Gravelliferone | 15.98 | 21316-80-3 | C_19_H_22_O_3_ |
| 128 | (4S,5S)-(+)-Germacrone 4,5-epoxide | 16.07 | 92691-35-5 | C_15_H_22_O_2_ |
| 129 | LysoPE(0:0/18:3(9Z,12Z,15Z)) | 16.51 | / | C_23_H_42_NO_7_P |
| 130 | LPI 18:3 | 16.70 | / | C_27_H_47_O_12_P |
| 131 | Citronellyl isovalerate | 16.74 | 68922-10-1 | C_15_H_28_O_2_ |
| 132 | Ginsenoside Re | 16.82 | 52286-59-6 | C_48_H_82_O_18_ |
| 133 | 6-O-Acetylaustroinulin | 16.92 | 75207-46-4 | C_22_H_36_O_4_ |
| 134 | 13-Cis-Acitretin | 17.10 | 69427-46-9 | C_21_H_26_O_3_ |
| 135 | Citronellyl hexanoate | 17.29 | 10580-25-3 | C_16_H_30_O_2_ |
| 136 | β-Linoleic acid | 17.33 | 60-33-3 | C_18_H_32_O_2_ |
| 137 | Pinolenic acid | 17.62 | / | C_18_H_30_O_2_ |
| 138 | 4'-O-Methylglabridin | 17.77 | 68978-09-6 | C_21_H_22_O_4_ |
| 139 | Gingerglycolipid B | 18.24 | 88168-90-5 | C_33_H_58_O_14_ |
| 140 | Pubescenol | 18.34 | 90685-93-1 | C_28_H_42_O_6_ |
| 141 | Ginsenoside Rg6 | 18.67 | 147419-93-0 | C_42_H_70_O_12_ |
| 142 | (20E)-Ginsenoside F4 | 18.80 | / | C_42_H_70_O_12_ |
| 143 | (R)-6'-O-(4-Geranyloxy-2-hydroxycinnamoyl)-marmin | 18.94 | 142628-36-2 | C_38_H_46_O_8_ |
| 144 | Tetracosatetraenoic acid | 21.52 | / | C_24_H_40_O_2_ |

* / indicating the related information is not acquired.
